# An Update on Postoperative Cognitive Dysfunction Following Cardiac Surgery

**DOI:** 10.3389/fpsyt.2022.884907

**Published:** 2022-06-15

**Authors:** Tony Vu, Julian A. Smith

**Affiliations:** ^1^Department of Cardiothoracic Surgery, Monash Health, Melbourne, VIC, Australia; ^2^Department of Surgery, School of Clinical Sciences at Monash Health, Monash University, Melbourne, VIC, Australia

**Keywords:** postoperative cognitive dysfunction, inflammation, cerebral hypoperfusion, impaired autoregulation, anaesthesia induced neurotoxicity, glycaemic control, intra-operative blood pressure management, near-infrared spectroscopy

## Abstract

Postoperative cognitive dysfunction is extremely prevalent following cardiac surgery. The increasing patient age and comorbidity profile increases their susceptibility to cognitive impairment. The underlying pathophysiological mechanisms leading to cognitive impairment are not clearly elucidated. Using the contemporary literature (2015–present), this narrative review has three aims. Firstly, to provide an overview of postoperative cognitive impairment. Secondly, to analyse the predominant pathophysiological mechanisms leading to cognitive dysfunction following cardiac surgery such as inflammation, cerebral hypoperfusion, cerebral microemboli, glycaemic control and anaesthesia induced neurotoxicity. Lastly, to assess the current therapeutic strategies of interest to address these pathophysiological mechanisms, including the administration of dexamethasone, the prevention of prolonged cerebral desaturations and the monitoring of cerebral perfusion using near-infrared spectroscopy, surgical management strategies to reduce the neurological effects of microemboli, intraoperative glycaemic control strategies, the effect of volatile vs. intravenous anaesthesia, and the efficacy of dexmedetomidine.

## Introduction

Over two million cardiac surgeries are performed globally per annum. Patients requiring cardiac surgery are of increasing age and are likely to possess comorbidities such as hypertension and diabetes mellitus. Elderly patients are at increased risk of post-operative complications, morbidity and mortality (1). Neurological sequalae are among the most prevalent complications following cardiac surgery. The American College of Cardiology and American Heart Association have classified postoperative neurological defects following cardiac surgery into Type I and Type II neuronal injury. Type I defects describe well-defined, focal brain insults such as those occurring from transient ischaemic attacks and stroke. On the contrary, Type II neuronal injury describes poorly understood brain insults such as those resulting in delirium or postoperative cognitive impairment (2). This narrative review aims to provide an overview of postoperative cognitive impairment, analyse the predominant pathophysiological mechanisms leading to cognitive dysfunction and assess the current therapeutic strategies of interest using the contemporary literature (2015–present).

### Definition

Postoperative cognitive dysfunction (POCD) is the most common neurological manifestation following cardiac surgery. Initially characterised by Bedford in 1955, POCD is generally defined as an impairment of cognitive functions such as executive function, attention, language, visuospatial interpretation, and motor skills (3, 4). Evered et al. (5) provided recommendations on the nomenclature of neurocognitive disorders (NCD) following surgery. These changes align with definitions in the Diagnostic and Statistical Manual of Mental Disorders 5th edition (DSM-5). NCDs are classified as mild or major in severity, with major NCDs resulting in significant cognitive impairment and disruptions to activities of daily living. The DSM-V criteria for the diagnosis of neurocognitive disorders are summarised below (6).

a) Subjective evidence of cognitive impairment.
  a. Either by the patient, clinician, or other informant.
b) Objective cognitive impairment.
  a. Mild: 1−2 standard deviations below average on neuropsychological batteries.
  b. Major: >2 standard deviations below average on neuropsychological batteries.
c) Disruptions to activities of daily living.
  a. Mild: No major disruption to capacity to perform activities of daily living.
  b. Major: Major disruption to capacity to perform activities of daily living.
d) The cognitive defects do not occur exclusively in the context of delirium.
e) Cognitive deficit is not better explained by another mental disorder.

Evered et al. (5) suggested that ‘perioperative NCDs’ should be used as an overarching term to include pre-existing cognitive impairment prior to surgery, acute events such as post-operative delirium, delayed neurocognitive recovery and post-operative mild/major (POCD) NCD. Delayed neurocognitive recovery is defined as cognitive impairment lasting up to 30 days post-operatively, even if the DSM-V criteria for a neurocognitive disorder are met. In contrast, post-operative mild/major NCD is defined as cognitive impairment persisting between 30 days and 1 year post-operatively. To reduce heterogeneity in future studies, POCD should refer to the definition of post-operative mild/major NCD as per Evered et al. (5). Lastly, cognitive impairment which persists for greater than 1 year following surgery lose the ‘postoperative’ specifier and are termed mild or major neurocognitive disorders as the impairment is unlikely to be linked to the occurrence of surgery (5). The distinction between delayed neurocognitive recovery and postoperative mild/major NCD (or POCD) manifest because recovery from the stressors of hospitalisation such as procedural insult, anaesthesia, sleep disturbance, medication side-effects largely occur within the 30 days (5). The distinction between postoperative delirium and POCD is difficult. It is not known whether they are entirely separate entities or lie on the same spectrum. Postoperative delirium is largely a clinical characterised by confusion and inattention. However, the diagnosis of postoperative delirium may be aided using the confusion assessment method (CAM). Conversely, POCD is characterised by changes to higher cognitive functioning, and its diagnosis requires the comparison of pre-operative and post-operative scores from neuropsychological testing (7, 8). This review will utilise the loose, clinical definition of POCD given that the nomenclature has not been widely adopted and the reviewed literature predominantly use this definition.

### Epidemiology

The incidence of POCD is unclear due to the wide range of criteria used to define cognitive dysfunction. Postoperative cognitive impairment affects between 10–40% of patients in the sixth postoperative week following cardiac surgery. Only around 45% of patients fully recover from cognitive impairment after 1 year post cardiac surgery (9). Furthermore, the presence of early cognitive impairment following surgery predicts the incidence and severity of cognitive dysfunction at 5 years following cardiac surgery (10).

### Risk Factors

The risk factors for POCD can be divided into pre-operative and intra-operative risk factors. The preoperative risk factors for POCD include advanced age (11–13), lower educational levels (12), pre-existing cognitive impairment (12, 14), APOE4 genotype carriers (15), depression (13), and diabetes (13, 16). The intraoperative risk factors include the surgical approach, use of cardiopulmonary bypass (CPB) (17), duration of surgery (13, 18) and anaesthesia (12, 13, 19), arterial pressure management (20), glycaemic management (21) and intraoperative haemoglobin concentration (18).

### Assessment of Postoperative Cognitive Dysfunction

The detection of POCD involves comparing pre-operative and post-operative neuropsychological test results (22). Due to variability in the tests used to detect POCD, a “Statement of Consensus” was produced to outline ideal conditions for measuring neuro-behavioural outcomes following cardiac surgery. The recommended core neuropsychological battery to assess for POCD is summarised in Table 1 (23). These tests were selected as they are easily administered, sensitive in detecting cognitive changes and can be standardised against normative data. Following cardiac surgery, memory, psychomotor speed function and attention are likely to be impacted and hence cognitive tests should be powered to detect these changes (24). Studies have also recommended the inclusion of mood assessment tools such as the Depression, Anxiety, and Stress Scale due to a potential association between mood and cognition (25). Despite the recommendations by the 1995 “Statement of Consensus,” a recent systematic review has found that those neuropsychological tests were performed in less than half of the sixty-two identified studies (26). Investigators select several neuropsychological tests which they believe to best examine the cognitive domains of interest. In several studies, investigators do not select a sufficient number of neuropsychological tests, or do not test all of the cognitive domains. Unfortunately, this practice has contributed to the heterogenous literature and impairs the robust comparison of studies examining POCD following cardiac surgery. The follow-up period for neuropsychological testing is also extremely variable between studies. This period is generally dictated by when the investigators believe they are likely to capture most cognitive impairment cases. The follow up period for these tests is also potentially limited by time, staffing, and financial constraints. Given the recent recommendations by Evered et al. (5), the follow up period in future studies should be up to a month post-operatively to detect cases of delayed neurocognitive recovery, and a year to detect postoperative mild/major neurocognitive disorders.

**TABLE 1:** Neuropsychological battery recommended by the 1995 “Statement of Consensus” for the assessment of POCD following cardiac surgery (23).

Neuropsychological Battery	Cognitive Domain Assessed
Trail Making Tests A	Attention and concentration
Trail Making Tests B	Executive functioning
Rey Auditory Verbal Learning Test	Verbal learning and memory
Grooved Pegboard	Motor skills

There are numerous neuropsychological tests which are used in assessing POCD.

#### Mini-Mental State Examination

The Mini-Mental State Examination (MMSE) is the most commonly used test in these studies, acting as a global test of cognition and a screening test for dementia. The test includes 30 questions, assessing attention, orientation, memory, language and visual-spatial skills. Importantly, the MMSE was not designed to detect POCD and should be used in conjunction with other neuropsychological tests. Scored between 0–30, a lower score indicates poorer performance. Traditionally, a score of 23 or 24 out of 30 is used as the cut-off score to define cognitive impairment, but this has not been validated (27).

#### Montreal Cognitive Assessment

The Montreal Cognitive Assessment (MoCA) is another commonly used bedside cognitive test, testing similar domains as the MMSE. The maximum test score is 30. A score of 26 and above is considered normal, whilst anything below this is considered cognitive impairment (28).

#### Rey Auditory Verbal Learning Test

The Rey Auditory Verbal Learning Test (RAVLT) assesses the ability to encode, store and retrieve verbal memory. In this test, a series of meaningful words are presented for five rounds. At the end of each round, immediate recall is tested by asking the participants to retrieve as many words as possible. Additionally, following the final round, delayed recall is tested by asking the patients to wait a period such as 20 min and attempt to retrieve as many words as possible (29).

#### Hopkins Verbal Learning Test

The Hopkins Verbal Learning Test (HVLT) assesses short term memory, verbal learning and delayed recall. The examiner reads twelve words aloud and asks the patient to recall as many as possible immediately afterwards. This is repeated three times. Subsequently, 20–25 min later, delayed recall is tested by asking the patient to recall as many as possible (30).

#### Trail Making Test

The Trail Making Test A (TMT-A) analyses psychomotor speed and attention by asking the patients to draw a trail, connecting numbered circles in ascending order without lifting their pencil off the paper. The Trail Making Test B (TMT-B) is similar to the TMT-A, but requires the patients to alternate between numbers and letters in ascending order. This added complexity assesses for executive functioning (31).

#### Concept Shift Test

The Concept Shift Test (CST) is similar to the TMT-A and TMT-B, assessing the patients’ ability to shift attention between stimuli. They are required to quickly cross out empty circles, or letters, numbers or a combination (32).

#### Wechsler Memory Scale

The Wechsler Memory Scale (WMS) has three subtests including the Visual memory Span, Digit Span Forwards and Digit Span backwards. The Visual Memory Span subtest assesses visual and short-term memory by showing patients three shapes for 10 s, taking them away and subsequently asking the patients to draw them. The Digit Span Forwards and backwards tests assess attention and short-term memory. The Digit Span Forwards subtest requires the participant to repeat a sequence of numbers read aloud by the investigator in the same order, whereas the Digit Span Backwards Subtest requires participants to repeat a sequence of numbers read aloud by the investigator in the reverse order (33).

#### Stroop Colour and Word Test

The Stroop Colour and Word test (SCWT) assesses the patients’ ability to inhibit cognitive interference during the processing of incongruent stimuli. A common example in practice involves giving the patient a list of words of names of colours. However, the list of words are printed in a colour which is different to the word itself. The examiner records the time spent reading the lists and the number of errors (34).

#### Grooved Pegboard Test

The Grooved Pegboard Test (GPT) is a complex manual dexterity test which assesses hand-eye coordination and psychomotor speed. The pegboard is a square apparatus containing 25 holes. Patients are required to pick up keyhole shaped pegs and manipulate them to fit into the holes. The time required for completion is recorded by the examiner. The patient may be asked to use only their dominant hand, non-dominant hand, or both (35).

### Neuropsychological Testing Limitations

Firstly, performing neuropsychological tests on unwell patients is difficult. Post-operatively, patients are recovering from surgical trauma, the effects of anaesthesia or medications, and complications are, therefore, unlikely to be able to cooperate with clinicians (25). Secondly, given the limited number of neuropsychologists available, clinicians often assess cognition using quick bedside tests such as the MMSE or MoCA (36). Whilst the use of these tests is acceptable, they should be complemented by other validated neuropsychological tests. Thirdly, neuropsychological tests should be adaptable to different languages as cultural differences and language barriers may impair the understanding of the test questions (37). Additionally, the MMSE and MoCA also require training of personnel to administer the neuropsychological tests. Moreover, since 2001, the use of the MMSE has been hindered by the implementation of copyright and usage fees (38). Furthermore, patients are often subjected to many neuropsychological tests. This may result in a patient becoming mentally fatigued throughout the tasks and performing more poorly in the later tasks. Conversely, patients undergoing neuropsychological testing may perform better during subsequent attempts due to the learning effect, where patients answer the test based on their previous experiences (39). Lastly, the neuropsychological test battery recommended by the “statement of consensus” are not universally adhered to according to Rudolph et al. (26). This impairs the ability to collate studies and perform meta-analyses which provide definitive conclusions.

### Statistical Assessment of Postoperative Cognitive Dysfunction

Numerous statistical methods have been used to assess POCD, contributing to the heterogenous literature. The analytic criteria used to define POCD include standard deviation (SD) changes, percentile changes, Z score changes, factor analysis and consensus of experts. Firstly, Standard deviation is typically used to measure the dispersion of data from the mean. Studies have defined POCD by a post-operative neuropsychological test result of 1 standard deviation below their baseline result. Results with an SD of 1.5 and 2 below the mean are defined as moderate and severe cognitive impairment respectively (40, 41). Secondly, Z scores are used to determine how far a score is from the mean. POCD can be defined as by a Z score greater than −1.96. The individual Z score is calculated by subtracting the mean score change between the pre-operative and post-operative tests in the control group from the difference in performance in the same tests in the surgical group. Then, this result is subsequently divided by the standard deviation of the mean score change in the control group (36, 42, 43). Thirdly, the percentile change criteria identify patients with POCD based on a specific decrease in the patients’ pre-operative and post-operative neuropsychological test scores. Ho et al. (44) and Keizer et al. (45) defined POCD as a decline of greater than 20% compared to their preoperative test results (44, 45). Moreover, the factor analysis technique utilises the standard deviation approach on a group of data derived from neuropsychological tests intending to analyse similar cognitive domains. Lastly, as the name suggests, the consensus of experts’ approach involves experienced clinicians determining whether a patient has POCD based on test results and clinical features.

### Implications of Postoperative Cognitive Dysfunction

Patients with POCD are more likely to have a slower recovery time, and an increased duration of hospital admission compared to patients without POCD (46). Additionally, these patients have an increased mortality rate in the year following their surgery (46). POCD is also associated with reduced adherence to rehabilitation programmes, potentially contributing to earlier retirement, increased morbidity and mortality. A 5-year prospective study revealed that neurocognitive function is strongly associated with quality of life and a patients’ perception of their own health. This is corroborated by evidence which suggests that postoperative cognitive changes following cardiac surgery predict cognitive deterioration 6 years following surgery (47–49). Furthermore, patients with higher cognitive function scores are more likely to productively contribute to society and maintain fulltime work (50). Evidence suggests that POCD may be associated with development of Alzheimer’s Dementia as anaesthesia may induce deposition of B-amyloid in the brain (51, 52). Thus, it is crucial to monitor and to optimise the pre- and post-operative management of cardiac surgery patients to reduce the likelihood of developing POCD and the potential long-term implications.

## Method

A literature search using OVID Medline and Embase was performed for this narrative review. To reflect the contemporary literature, the included studies were published between 2015 and present. However, for background and contextual information, this limit was removed. Additional relevant articles, such as novel pre-clinical studies examining pathophysiological mechanisms leading to POCD, were obtained using references from the identified articles. Keywords for POCD included POCD; postoperative cognitive dysfunction; cognitive impairment; neuronal injury; neuroinflammation; neurocognitive test; neurocognitive battery; rey auditory verbal learning test; grooved pegboard; trail making test; neuropsych*; neurocog*; cognitive test; cognitive performance and cognitive dysfunct*. The following keywords for cardiac surgery were included: cardiac surgery; heart surgery; cardiac operation; cardiothoracic surgery; coronary artery bypass graft; CABG; revascularisation surgery; valve replacement and valve repair. Limits included English language, human studies and adults only. As mentioned previously, the loose/old definition of POCD is used given that minimal studies have adopted the 2018 nomenclature recommendations.

**FIGURE 1:**
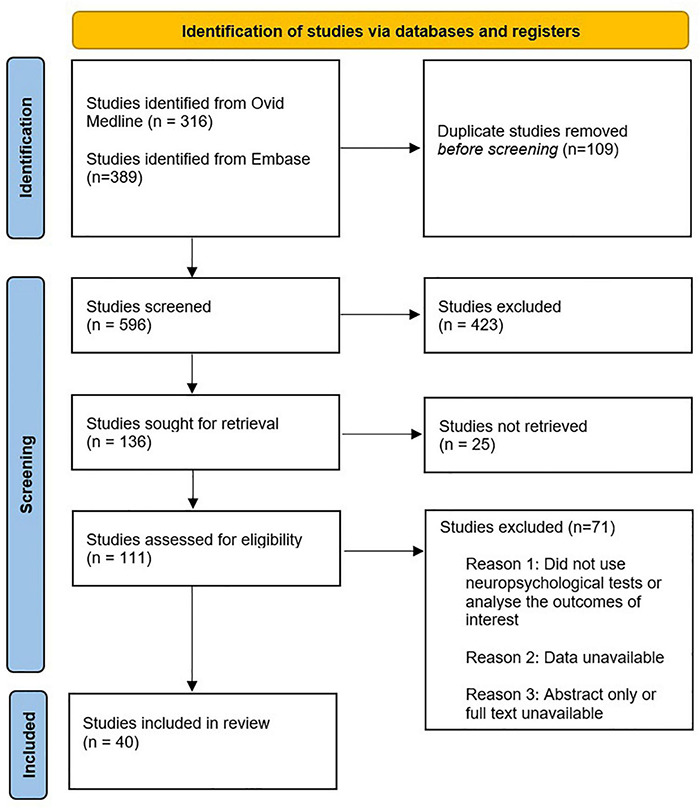
The identification and selection of studies included in this narrative review.

The identification and selection of studies is depicted in Figure 1. Study designs of interest included cohort studies, randomised controlled trials and systematic reviews. Systematic reviews were not included in the table of studies to avoid duplication with other studies. However, they were discussed in the main text given they are a high level of evidence. Forty studies were discussed in this narrative review. This compromises 4 studies on dexamethasone, 4 studies on autoregulation/intraoperative blood pressure management, 10 studies on the use of NIRS to detect cerebral desaturations, 5 studies on micro-emboli, 5 on intra-operative glycaemic control, 6 studies on anaesthesia induced- neurotoxicity and 6 studies on dexmedetomidine.

## Pathophysiology of Postoperative Cognitive Dysfunction Following Cardiac Surgery

**FIGURE 2:**
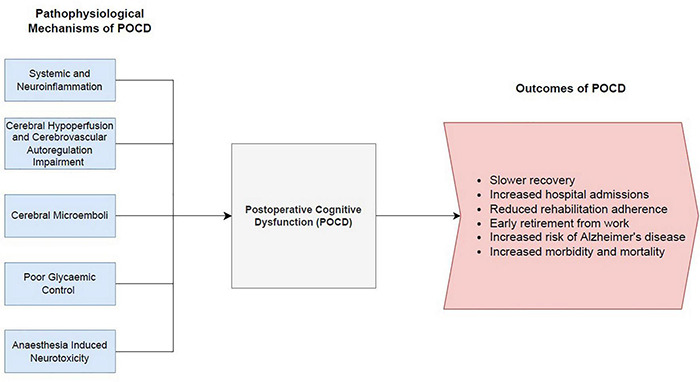
Summary diagram of the pathophysiological mechanisms leading to post-operative cognitive dysfunction and the resulting outcomes of POCD.

It was previously thought that POCD following cardiac surgery was predominantly driven by an inflammatory response due to cardiopulmonary bypass. However, off-pump cardiac surgery studies have yet to convincingly demonstrate a reduction of cognitive impairment compared to traditional on-pump cardiac surgery (53, 54). Consequently, there is significant interest in understanding the underlying mechanisms contributing to POCD. There are numerous proposed pathophysiological mechanisms leading to the development of POCD. This review will focus on several main theories, including systemic and neuroinflammation, cerebral hypoperfusion and cerebrovascular autoregulation impairment, cerebral microemboli, impaired glycaemic control and anaesthesia induced neurotoxicity. A summary diagram of the mechanisms leading to POCD, and the resulting implications of POCD is depicted in Figure 2.

### Inflammation

#### Systemic and Neuroinflammation

The link between inflammation and POCD is complex. There are several activators of the inflammatory response in cardiac surgery including surgical trauma, blood surface interactions with CPB circuitry and ischaemic reperfusion injury (55). Firstly, surgical trauma results in the release of danger associated molecular patterns (DAMPs) such as high-mobility group 1 (HMGB1) and S100A8. HMGB1 is associated with parenchymal neuroinflammation and neurocognitive impairment (56, 57). This is supported by the reduction in POCD and memory defects following the use of HMGB1 inhibitors in rodent models following liver surgery (58). In contrast, S100A8 is associated with macrophage migration into the brain and is likely to play a key role in POCD. Pre-operative administration of an S100A8 inhibitor in mice improved cognitive functioning 24 h following surgery and inhibited hippocampal gliosis (59). DAMPs bind to pattern recognition receptors on the endothelium to activate the transcription factor nuclear factor kappa beta (NF-KB), triggering a pro-inflammatory cascade. The pro-inflammatory cascade involves the release of cytokines such as interleukin-1, interleukin-6, and tumour necrosis factor-a (TNF-a), and upregulates the expression of cyclooxygenase-2 (COX2). COX2 is implicated in the disruption of the blood brain barrier (BBB) by upregulating the expression of matrix metalloproteinases (MMP), contributing to neuroinflammation (60). Secondly, blood exposure to the CPB circuitry causes systemic inflammation, involving both the humoural and cellular response (61). Adsorption of the C3 complement protein to the circuitry activates the alternative complement pathway. The formation of heparin-protamine complexes at the completion of surgery activates the classical complement pathway (55). Increased inflammatory markers within the periphery or CNS are associated with the development of POCD (62–67). Furthermore, Zhu et al. (65) demonstrated a significant association between increased TNF-a levels and decreased executive functioning (65). However, Nemeth et al. did not detect an association between magnitude of the inflammatory response and incidence of POCD (68).

#### Blood Brain Barrier Disruption

The blood brain barrier consists of endothelial cells, basement membrane and glial cells, functioning to limit the entry of deleterious substances into the brain (69). Cardiac surgery has been implicated in the development of BBB disruption, with the extent of the disruption correlating with the magnitude of POCD (70). BBB dysfunction leads to potential exposure to toxic and pro-inflammatory substrates. One theory explaining this disruption suggests that microembolisation triggers an interaction with the vascular endothelium, impairing the BBB (71). However, as previously stated, it is believed that the pro-inflammatory response triggers the activation of MMPs which proteolytically degrade the BBB (72, 73). The use of CPB in porcine models increases the permeability of the BBB (74). Merino et al. (75) demonstrated that following human cardiac surgery, BBB disruption is most prevalent within the first 24 h post-operatively (75). Abrahamov et al. (76) demonstrated a relationship between on-pump CABG and BBB disruption. MRI studies demonstrated that the BBB disruption was predominantly localised to the frontal region of the brain. This disruption correlates with a significant decrease in attention and executive functioning postoperatively (76). Similarly, Danielson et al. (71) showed that on-pump CABG triggers BBB dysfunction as well as systemic and neuroinflammation (71). Following the disruption of the BBB, pro-inflammatory cytokines such as TNF-a pass through the BBB, and there is an upregulation of chemokines such as MCP-1 which recruit bone marrow derived macrophages into the CNS (77, 78). Subsequently, the bone-marrow derived macrophages secrete further pro-inflammatory cytokines, reactive oxygen species, and activate microglia cells (79). Microglial cells may adopt an inflammatory phenotype which further contributes to neuroinflammation and cognitive impairment (80).

#### Dexamethasone to Attenuate the Inflammatory Response

Given the link between inflammation and POCD, the use of glucocorticoids such as dexamethasone to attenuate the inflammatory response has been proposed. Dexamethasone has a long duration of action and may reduce neuroinflammation and cerebral oedema (81). However, prolonged use of dexamethasone is itself associated with cognitive impairment (82). Results from recent studies are conflicting and are summarised in Table 2. Four studies were identified. Glumac et al. (63) found that 0.1 mg/kg of dexamethasone reduced the incidence of POCD within the first 6 days compared to placebo (RR: 0.43; 95% CI: 0.21–0.89; *p* = 0.02) (63). Glumac and colleagues also demonstrated that patients receiving dexamethasone performed significantly better in the MMSE, symbol digit modality test and simple reaction time tests compared to the placebo group at POD6. This suggests that dexamethasone may preserve global cognition and positively influence psychomotor processing speed. That was also the first study to confirm that the systemic inflammatory response is inhibited by dexamethasone based on inflammatory markers (*p* < 0.001). In a follow-up from their 2017 study, Glumac et al. (83) observed that dexamethasone reduced the incidence of cognitive impairment over 4 years. However, this finding narrowly failed to reach significance (RR: 0.459; 95% CI: 0.192–1.100; *p* = 0.068) (83). This study was the first long-term study to assess the effect of dexamethasone on cognitive outcomes following cardiac surgery. The 4-year timepoint for post-operative follow up was chosen as it was likely to truly reflect the long-term effect of dexamethasone prior to surgery. An earlier post-operative follow-up point, such as at 1 year postoperatively, was not chosen because it was unlikely to reflect the long-term effect of dexamethasone. Conversely, follow up after 5 years postoperatively may also not reflect the effects of dexamethasone, as the results may be confounded by age related cognitive decline, the development or progression of new cardiovascular or cerebrovascular disease, and the development of dementia. Glumac et al. (83) also investigated whether cognitive impairment at POD6 influenced the development of cognitive impairment at 4 years. Thirteen patients who did not have POCD at POD6, had POCD detected at 4 years post-operatively. Ten of these thirteen patients were in the placebo group. These results reveal that dexamethasone may attenuate the inflammatory response and potentially prevent long term cognitive impairment.

**TABLE 2:** The effect of dexamethasone on POCD.

Study	Aim	Surgery Type	Study Type	Patient number	Definition of POCD	T1 Cognitive Assessment	T2 Cognitive Assessment	Psychological Assessment Tool	Findings
Sauer et al. (84)	To determine whether the intraoperative administration of high dose dexamethasone would reduce delirium during the first 4 postoperative days	On-pump cardiac surgery	RCT Dexamethasone 1 mg/kg vs. placebo administer at the induction of anaesthesia	737 Dexamethasone (*n* = 367) Placebo (*n* = 370)	Not specified	Not specified	POD1-4	CAM	Intraoperative administration of dexamethasone did not significantly reduce the incidence of delirium (OR: 0.85, 95% CI: 0.55–1.31) Incidence of delirium in dexamethasone vs. placebo group: 14.2 vs. 14.9%

Glumac et al. (63)	To determine whether prophylactic dexamethasone reduces POCD	Cardiac surgery	RCT Dexamethasone (0.1 mg/kg) vs. placebo administered 10 h prior to surgery	161 Dexamethasone (*n* = 80) Placebo (*n* = 81)	RCI ≤ 1.96 on at least one test	Two days before surgery	POD 6	MMSE RAVLT WMS (Involving subtest of Visual Memory Span, Digit Span Forward, Digit Span Backward) SDMT PsychE	The dexamethasone group had a reduced incidence of POCD compared to placebo (RR: 0.43, 95% CI: 0.21–0.89, *p* = 0.02). Patients in the dexamethasone group performed significantly better in the MMSE, Symbol digit modality test and simple reaction times compared to placebo group at day six (*p* < 0.05). Dexamethasone also reduced systemic inflammatory response compared to placebo (30 vs. 58%; *p* < 0.001).

Glumac et al. (83)	Four years follow study to determine whether prophylactic dexamethasone reduced POCD	Cardiac surgery	Follow up of previous RCT Dexamethasone (0.1 mg/kg) vs. placebo administered 10 h prior to surgery	116 Dexamethasone (*n* = 54) Placebo (*n* = 62)	RCI ≤ 1.96 on at least one test	Two days before surgery	POD 6 and 4 years postoperatively	MMSE RAVLT WMS PsychE	Dexamethasone did not significantly reduce POCD at 6 days (RR: 0.510; 95% CI: 0.241–1.079; *p* = 0.067) Dexamethasone did not significantly reduce POCD at 4 years (RR: 0.459; 95% CI: 0.192–1.100; *p* = 0.068)

*CABG, Coronary Artery Bypass Graft; CAM, confusion assessment method; MMSE, mini mental state exam; POCD, postoperative cognitive dysfunction; RAVLT, rey auditory verbal learning test; RCT, randomised control trial; SDMT, symbol digit modalities test; T1, pre-operative timepoint for neurocognitive testing; T2, post-operative timepoint for neurocognitive testing; WMS, wechsler memory scale test.*

The results of Sauer et al. (84) and Li et al. (85) did not support the use of dexamethasone to reduce cognitive impairment. Sauer et al. demonstrated that the intraoperative administration of 1 mg/kg dexamethasone did not reduce delirium during the first four postoperative days (84). Similarly, a meta-analysis by Li et al. (85) involving 1460 patients did not support the use dexamethasone compared to placebo to reduce POCD (RR: 1.00; 95% CI: 0.51–1.96). A subgroup analysis of cardiac surgeries was also non-supportive (RR: 0.90; 95% CI: 0.21–3.77) (85). These conflicting results may be caused by variation in the dose of dexamethasone used. As previously mentioned, high doses of dexamethasone, such as those used by Sauer, may contribute to cognitive impairment as the hippocampus has a high concentration of glucocorticoid receptors, and hence yield negative results. Moreover, heterogeneity in the timing of dexamethasone administration may cause conflicting results. Glumac, who demonstrated positive benefits, administered dexamethasone 10 h prior to surgery as they believed this would allow sufficient time for the anti-inflammatory properties to work (63). In contrast, the negative studies administered dexamethasone at the induction of anaesthesia (84, 85). Further studies are required before a definitive conclusion can be made regarding the efficacy of dexamethasone on cognitive impairment following cardiac surgery.

### Cerebral Hypoperfusion

#### Cerebrovascular Autoregulation

Cerebrovascular autoregulation refers to the ability of the brain to maintain stable cerebral perfusion *via* myogenic, neurogenic, and metabolic mechanisms despite changes in cerebral perfusion pressure (86). The cerebral perfusion pressure is calculated as the difference between mean arterial pressure (MAP) and the larger of either central venous pressure or intracranial pressure. It is generally accepted that this autoregulatory mechanism functions optimally between a MAP of 60–160 mmHg (87). If the MAP is above the higher limit of the autoregulation zone, there is excessive cerebral blood flow, leading to oedema and capillary damage. Conversely, if the MAP is below the lower limit of the autoregulatory zone, cerebral ischaemia ensues (88). Fluctuations in cerebral perfusion are common during cardiac surgery and may fall below levels necessary to maintain autoregulation (89, 90).

Kumpaitiene et al. (91) aimed to detect episodes of impaired cerebrovascular autoregulation and determine whether there was an association with POCD following cardiac surgery. They assessed cerebrovascular autoregulation using a novel, non-invasive ultrasonic monitor known as the ‘Vittamed 505 Monitor.’ POCD was present in twenty-two patients (37%), with a significant difference between the groups in the attention test (*p* = 0.0016), Rey Auditory Visual Learning Test (*p* = 0.04) and digit symbol substitution test (*p* = 0.005). Impaired cerebrovascular autoregulation was detected in all 59 patients, with a mean duration of autoregulatory impairment being 4.9 min. Building on this, they found that the mean duration of the longest cerebrovascular episode was significantly longer in patients experiencing POCD (6.38 min) compared to patients without POCD (4.22 min, *p* = 0.001). Furthermore, Kumpaitiene and colleagues determined that 15 of their patients had a lower cerebrovascular autoregulatory limit between 61.4–69.5 mmHg (91). Traditionally, cardiac surgeons have maintained an intraoperative MAP between 50–60 mmHg as it was believed that this was the lower autoregulatory limit (92). Hence, aiming for a low intraoperative blood pressure in these patients may cause end-organ damage and cognitive impairment. Thus, it is ideal that future studies confirm the association between cerebrovascular autoregulation impairment and POCD with larger multi-centred studies and consider integrating the identification of patient specific cerebrovascular autoregulatory limits into clinical practice.

#### Intraoperative Blood Pressure Management

Two randomised controlled trials and a meta-analysis was identified examining the effect of high vs. low intraoperative blood pressure management on post-operative cognitive impairment following cardiac surgery (Table 3). Overall, there is conflicting evidence on the best target for MAP to reduce POCD following cardiac surgery. Vedel et al. (93) conducted the Perfusion Pressure Cerebral Infarcts trial to determine the effect of a higher vs. lower blood pressure target by titrating using noradrenaline during on-pump cardiac surgery on cerebral injury. Patients were allocated to either a higher MAP target (70–80 mmHg), or a lower MAP target (40–50 mmHg) (93). They detected the number and volume of new cerebral lesions using diffusion weighted MRI. Vedel and colleagues found that targetting higher blood pressures during on-pump surgery did not affect the volume of new cerebral lesions as detected by diffusion weighted MRI. The low MAP target group had a median cerebral infarct volume of 25 mm^3^ (Interquartile Range: 0–118), whilst the high MAP target group had a median cerebral infarct volume of 29 mm^3^ (Interquartile Range: 0–143). New brain lesions were present in 52.8 and 55.7% of patients in the low and high MAP target group respectively. There was no difference in the incidence of POCD between the low and high MAP groups at discharge (OR = 1.76, 95% CI: 0.90–3.47, *p* = 0.12). Additionally, there was no difference in the incidence of POCD between low and high MAP groups at the second post-operative follow up (OR = 0.72, 95% CI = 0.23–2.31, *p* = 0.77), with a median follow up time of 3 months. Notably, there was a trend towards significance in the occurrence of strokes in the higher MAP group compared to the low MAP group (6 vs. 1.1%, *p* = 0.06). This study has strongly contributed to the 2019 guidelines released by the European Association for Cardio-Thoracic Surgery which currently recommend targetting an intraoperative MAP between 50–80 mmHg (94). Moreover, this study highlights the potential of using diffusion weighted MRI to supplement baseline cognitive testing. Larsen et al. (95) conducted a 3-year follow-up study of the Perfusion Pressure Cerebral Infarcts trial (95). They found no difference in mortality over a median follow up period of 3.4 years between the high and low MAP target groups (HR = 1.23, 95%CI: (0.33–3.12), *p* = 0.65). Furthermore, there was no difference in the development of POCD between the high and low MAP target groups [OR = 1.01, 95% CI: (0.33–3.12)]. Kiabi et al. (96) performed a meta-analysis to determine the effect of intraoperative low vs. high MAP on the development of POCD following on-pump CABG. They identified three studies which were not previously examined in this review, including Gold et al. (97), Charlson et al. (98) and Siepe et al. (99). From a patient population of 731, the intraoperative maintenance of low MAP did not reduce the incidence of POCD compared to a higher intra-operative MAP (RR = 1.012, 95% CI: [0.277–3.688], *p* = 0.986). However, it was noted that a shorter cardiopulmonary bypass time was associated with a lower incidence of POCD, regardless of which group the patients were allocated to (RR-logarithmic form = −0.519, 95% CI: [−0.949, −0.089], *p* = 0.017) (96). These results suggest that perhaps the longer bypass time did potential benefits of a low intraoperative MAP on post-operative cognitive function to be observed.

**TABLE 3:** Intraoperative blood pressure management and cerebrovascular autoregulation.

Study	Aim	Surgery Type	Study Type	Patient Number	Definition of POCD	T1 Cognitive Assessment	T2 Cognitive Assessment	Psychological Assessment Tool	Findings
Vedel et al. (93)	To determine the effect of a higher vs. lower blood pressure target as titrated using noradrenaline during on-pump cardiac surgery on cerebral injury	On-pump CABG and/or left sided heart valve surgery	RCT (PPCI Trial) Higher MAP Target (70–80 mmHg) Lower MAP Target (40–50 mmHg)	169 High target (*n* = 98) Low target (*n* = 99)	Combined Z score > 1.96 from neuropsychological tests	Day before surgery	At discharge from hospital or 1 week (whichever was earlier) Second follow up was between 2–4 months postoperatively	VLT CST SCWI PPMST LDC Four boxes test	Targetting higher blood pressures during on-pump surgery did not affect the volume or number of cerebral infarcts detected by diffusion weighted MRI. Brain lesions were present in 52.8 and 55.7% of patients in the low and high MAP target group respectively No difference in the incidence of POCD between the low and high MAP groups at discharge (OR = 1.76, 95% CI: 0.90–3.47, *p* = 0.12) No difference in the incidence of POCD between low and high MAP groups at second follow up (OR = 0.72, 95% CI = 0.23–2.31, *p* = 0.77). Median time for second follow up was 92 days post-operatively

Kumpaitiene et al. (91)	To detect episodes of impaired cerebrovascular autoregulation and the association between this impairment and POCD	On-pump CABG	Prospective observational study Cerebrovascular autoregulation assessed using non-invasive ultrasonic monitor (Vittamed 505 monitor)	59	Patients’ combined score >2, or at least two scores for separate tests were >2 Combined scores were calculated by dividing the sum of all individual scores by the SD of the mean sum of all the patients’ scores	Day before surgery	POD10	MMSE RAVLT WAIS (Digit Span and Digit Symbol Substitution) Schulte table	Incidence of POCD was 37% (22 patients) All patients experienced impaired cerebrovascular autoregulation Mean duration of cerebrovascular autoregulatory impairment was 4.9 min Duration of the single longest cerebrovascular autoregulation impairment was associated with the incidence of POCD. Mean duration of the longest cerebrovascular autoregulatory impairment episode was significantly longer in the POCD group (6.38 min) compared to non-POCD group (4.22 min) (*p* = 0.001).
Larsen et al. (95)	As a follow up study to the PPCI trial, they investigated whether patients allocated to a higher MAP target had a higher long term mortality at 3 years follow up	On-pump CABG and/or left sided heart valve surgery	Follow up observational study to PPCI Trial Higher MAP Target (70–80 mmHg) Lower MAP Target (40–50 mmHg)	113 patients followed up High target (*n* = 55) Low target (*n* = 58)	Combined Z score >1.96 from neuropsychological tests	Day before surgery	At discharge from hospital or 1 week (whichever was earlier) Second follow up was between 2–4 months postoperatively Final follow up was at 3 years	VLT CST SCWI PPMST LDC Four boxes test	No difference in mortality over a median follow up period of 3.4 years between the high and low MAP target groups (HR = 1.23, 95% CI: (0.33–3.12), *p* = 0.65) No difference in the development of POCD between the high and low MAP target groups [OR = 1.01, 95% CI: (0.33–3.12)]

*CABG, coronary artery bypass graft; CST, concept shifting test; LDC, letter digital coding; MAP, mean arterial pressure; MoCA, montreal cognitive assessment; MMSE, mini mental state exam; POCD, postoperative cognitive dysfunction; POD, postoperative day; PPCI, perfusion pressure cerebral infarct; PPMST, paper and pencil memory scanning test; RAVLT, rey auditory verbal learning test; RCT, randomised control trial; SCWI, stroop colour word interference; SDMT, symbol digit modalities test; T1, pre-operative timepoint for neurocognitive testing; T2, post-operative timepoint for neurocognitive testing; VLT, verbal learning test; WMS, wechsler memory scale test.*

#### Monitoring of Cerebral Perfusion *via* Near-Infrared Spectroscopy

The monitoring of cerebral perfusion is an area of interest to reduce POCD. Regional cerebral oxygenation may be non-invasively monitored using near-infrared spectroscopy (NIRS). This involves attaching probes, one transmitter and two sensors, onto the skin of the patients’ forehead (100). One sensor detects the absorption of infrared light from superficial tissue, whilst the other detects infrared light from deeper tissue (100). Subsequently, the transmitter emits infrared light, which is absorbed by haemoglobin. Oxygenated and deoxygenated haemoglobin absorb infrared light differently, allowing real-time monitoring of regional cerebral oxygenation by comparing the balance between cerebral oxygen consumption and supply (101, 102). NIRS does not distinguish between arterial and venous blood but reflect the overall regional balance of oxygen supply and demand. This is known as regional cerebral oxygen saturation (rSO2). In contrast, pulse oximetry aims to determine the saturation of oxygen in arterial blood (103). Monitoring of rSO2 with NIRS allows for a rapid correction of cerebral desaturations using a standardised protocol. This involves reducing mechanical obstruction to cerebral flow by repositioning of the head or venous canulae, increasing cerebral oxygen delivery (increasing FiO2, pCO2 or PaCO2, red blood cell transfusions or pump flow rate), or reducing oxygen consumption (increasing depth of anaesthesia or hypothermia) (104).

The efficacy of detecting cerebral desaturations using NIRS to prevent POCD following cardiac surgery is unclear. Ten studies were identified, with the results summarised in Table 4. Five studies supported the use of NIRS for the early detection of cerebral desaturations to prevent POCD. Kara et al. (105) demonstrated that the use of NIRS during cardiac surgery to detect desaturations of rSO2 > 20% is associated with an increase in MoCA scores post-operatively (*p* < 0.001) (105). The standardised protocol mentioned previously was applied when cerebral desaturations were greater than 20%. Mild cognitive impairment was present in 16.3% of patients in the NIRS group, whilst it was present in 44.4% of the non-NIRS group. The use of NIRS as a cerebral oximeter was also correlated with an increase in MoCA scores (*r* = 0.59, *p* < 0.001). Similarly, Colak et al. (106) demonstrated that the incidence of POCD was significantly less in the NIRS group compared to the control group (*p* = 0.02). Additionally, they found that prolonged rSO2 desaturations were the strongest predictor of cognitive decline (106). Zorilla et al. conducted a meta-analysis and found that the use of intraoperative NIRS is associated with a significant reduction in POCD at 1 week postoperatively (RR 0.55; 95% CI 0.36–0.86; *p* = 0.009) (107). Uysal et al. (108) used intraoperative NIRS monitoring to detect episodes of rSO2 < 60% for more than 60 s. Upon reaching this threshold, the previously mentioned standard intervention was applied to increase rSO2 until it was at least 60% on both probes. They found that patients allocated to the NIRS group had significantly better memory scores at the 6-month post-operative follow up compared to the control group (0.60 vs. −0.17; *p* = 0.008). However, there was no difference in response speed, processing speed or attention at the 6 month follow up. They also did not find an association between the duration of and severity of the cerebral desaturations with the development of POCD (108). Furthermore, Soenarto et al. (103) conducted a cohort study and demonstrated that the episodes of cerebral desaturation of >20% were significantly longer in patients with POCD (*p* = 0.007) (103). However, the magnitude of rSO2 desaturations was not associated with POCD.

**TABLE 4:** Studies examining the efficacy of NIRS to detect cerebral desaturations in preventing POCD following cardiac surgery.

Study	Aim	Surgery Type	Study Type	Patient number	Definition of POCD	T1 Cognitive Assessment	T2 Cognitive Assessment	Psychological Assessment Tool	Findings
Kara et al. (105)	To investigate whether intraoperative cerebral oximetry using NIRS should be used to prevent POCD following CABG	On-pump CABG	RCT NIRS vs. No NIRS A standardised desaturation algorithm was commenced when rSO2 value dropped by more than 20% compared to baseline	79 NIRS (*n* = 43) No NIRS (*n* = 36)	MoCA Score: <26 Mild cognitive impairment: 19–25 Serious cognitive impairment: <19	Before surgery	Not specified	MoCA	The NIRS group had 16.3% patients with mild cognitive impairment, whilst 44.4% of non-NIRS group had mild cognitive impairment (*p* = 0.01). Use of NIRS is with a correlated increase in MoCA score (*r* = 0.59, *p* < 0.001) Post-operative MoCA score in NIRS group was 26.8 ± 1.9, whilst non-NIRs group was 23.6 ± 2.5, *p* < 0.001. Statistically significant decrease in mean postoperative MoCA score compared to baseline in no NIRS group

Colak et al. (106)	To determine whether cerebral oximetry monitoring (INVOS) can decrease POCD following CABG	On-pump CABG	RCT NIRS vs. control Intervention group maintained rSO2 > 80% of baseline or >50% absolute value. If rSO2 decreased below these values, standardised protocol commenced	200	Decrease in performance on at least one test	Day before surgery	POD7	MMSE CTT1 GPT	POCD Incidence: 40.3% POCD incidence was significantly less in the INVOS NIRS group (28%) compared to control group (52%; *p* = 0.02) at POD7. Prolonged rSO2 desaturation was the strongest predictor of cognitive decline in INVOS group

Dubovoy et al. (49)	To determine whether management of cerebral oximetry desaturations is associated with improved cognitive outcomes	Aortic reconstruction under deep hypothermic circulatory arrest	Retrospective analysis	17	Decrease in summed normalised score from neuropsychological battery compared to baseline	Not specified	3 months postoperatively	HVLT leaning/delay/ discrimination index COWAT BVMT delay/recognition discrimination	POCD in 17 (41%) of patients No association between baseline cerebral oximetry and cognitive dysfunction “POCD is associated with loss of complexity of the time series as shown by a decrease in FwFn”
Rogers et al. (113)	To assess whether personalised optimisation of cerebral perfusion using NIRS would reduce perioperative brain, kidney and heart injury.	Combined on-pump CABG+ valve surgery, isolated valve surgery.	RCT Personalised vs. generic algorithms for optimising tissue oxygenation The generic algorithm optimised tissue oxygenation using measures of oxygen utilisation and a predefined intraoperative haematocrit transfusion threshold of 23% NIRS algorithm aimed to maintain an absolute rSO2 value >50%, or >70% compared to baseline. A restrictive intraoperative haematocrit transfusion threshold of 18% was applied	204 Patient specific algorithm (*n* = 98) Generic Algorithm (*n* = 106)	Not specified	Pre-operatively	POD4-7, 3 months	TMT A + B RAVLT WIAS (Digit Symbol Test) COWAT GPT MMSE Anxiety/depression	NIRS group performed better for verbal fluency (non-core cognitive domain; mean difference 3.73; 95% CI: 1.50, 5.96) No difference between groups for core cognitive domains (attention, verbal memory, motor coordination) and non-core cognitive domains (psychomotor speed and visuospatial skills)

Kumpaitiene et al. (109)	To determine the correlation between decreased rSO2 saturation, POCD and blood levels of brain injury markers following cardiac surgery	On-pump CABG	Prospective observational trial Cerebral desaturation was defined as a decrease in rSO2 ≥ 20% from baseline or an absolute rSO2 of ≤45%	59	Sum of Z score > 2, or at least two Z scores for separate tests > 2	Eve of surgery	POD7-10	MMSE RAVLT TMT A + B WIAS (Digit Span and Digit Symbol Substitution Test) Schulte table	POCD in 22 (37%) patients Decreased rSO2 in 21 (35%) patients Short term episodes of decreased rSO2 during surgery is not correlated with incidence of cognitive impairment No significant changes in the level of glial fibrillary acidic protein following surgery. Neuron-specific enolase increased in 29 (49%) of patients following surgery, but there was no association with POCD

Holmgaard et al. (111)	To identify whether longer cumulative times of rSO2 > 10% below baseline as detected using NIRS is associated with POCD	On-pump CABG	Secondary analysis of parallel group randomised trial	148	Sum of Z score > 2, or at least two Z scores for separate tests > 2	Day before surgery	First follow up was day before discharge or POD8, whichever came first Second follow up was 3 months later	ISPOCD battery (VLT, CST, SCWI, and LDC)	44 (29%) of patients had POCD at discharge, 12 (8%) of patients had POCD at 3 months The median time of rSO2 > 10% below baseline did not differ between patients with or without POCD at discharge (Difference = 0.0 min, 95% CI: (−3.11, 1.47), *p* = 0.88) No significant association between any neuropsychological test outcomes and POCD No significant association between intra-operative rO2 and incidence of POCD

Uysal et al. (108)	Assess whether optimising cerebral oxygenation using intraoperative NIRS is associated with better neurocognitive outcomes compared to current care	On-pump cardiac CABG or valve surgery	RCT NIRS intervention vs. control Intraoperative rSO2 < 60% for more than 60 s triggered intervention Intervention protocol initiated until rSO2 was restored to at least 60% on both probes	125 NIRS Group (*n* = 59) Control group (*n* = 66)	Change in 4 cognitive factor scores from preoperative to postoperative time points	>24 h before surgery	3 and 6 months postoperatively	Cognitive Stability Index Battery (10 subtests which analyse response speed, processing speed, attention and memory)	Intraoperative NIRS triggering early intervention resulted in significantly better memory scores at the 6-month postoperative follow up (0.60 vs. −0.17; *p* = 0.008). No difference in response speed, processing speed, or attention at 6 months. However, duration and severity of desaturation was not associated with cognitive impairment.

Bennett et al. (101)	To determine whether their NIRS protocol would improve neurological outcomes postoperatively prior to discharge and at 6 months postoperatively	Cardiac surgery with CPB	Double blinded RCT Intervention initiated if rSO2 dropped below baseline on room air	182 NIRS Group (*n* = 91) Conventional management group (*n* = 91)	Not specified	Day before surgery	POD3-5 and 6 months	MMSE HVLT TMT A/B Anti-saccadic eye test	Significant improvement in self-reported 6-month general functioning in the NIRS group (*p* = 0.016) No difference between groups in neurocognitive tests (*p* = 0.498) and neurological dysfunction (*p* = 0.600) postoperatively Duration of ICU stay was significantly shorter in NIRS group (*p* = 0.026)
Semrau et al. (114)	To determine the relationship between rSO2 and postoperative neurological function	On-pump CABG	Prospective observational trial	40	Z score > 1.96	Day before surgery	3 months following surgery	RBANS and KINARM (robotic technologies)	Pre-operative cognitive impairment accounted for 82.2% of variance in post-operative performance
Soenarto et al. (103)	To determine the relationship between POCD and rSO2 using NIRS	Cardiac surgery with CPB	Prospective observational trial Desaturation defined as >20% decrease on NIRS compared to baseline	70	>20% drop from baseline on at least 2 cognitive tests	Day before surgery	POD5	RAVLT TMT Digit Span (forwards and backwards)	POCD incidence: 51.7% (31 patients) Total cerebral desaturation was significantly longer in patients with POCD (*p* = 0.007) Absolute value of rSO2 was not associated with POCD but a drop in rSO2 > 20% from baseline may be associated with POCD

*BVMT, brief verbal memory test; CABG, coronary artery bypass graft; COWAT, controlled oral word association test; CST, concept shifting test; CTT1, colour trail test 1; GPT, grooved pegboard test; HVLT, hopkins verbal learning test; LDC, letter digit coding; MMSE, mini-mental state examination; MoCA, montreal cognitive assessment scale; NIRS, near infrared spectroscopy; POCD, postoperative cognitive dysfunction; POD, postoperative day; RAVLT, rey auditory verbal learning test; RCT; randomised control trial; SCWI, stroop colour word interference; rSO2, regional cerebral oxygen saturation; T1, pre-operative timepoint for neurocognitive testing; T2, post-operative timepoint for neurocognitive testing; TMT, trail making test; VLT, verbal learning test; WIAS, wechsler adult intelligence scale.*

Five studies did not show the clear benefit of detecting cerebral desaturations with NIRS and providing early intervention in preventing POCD following cardiac surgery. In a study of 59 patients, Kumpaitiene et al. (109) aimed to demonstrate an association between a reduction in rSO2, POCD and the level of brain injury markers. They defined cerebral desaturation as a decrease in decrease in rSO2 ≥ 20% from baseline or an absolute rSO2 of ≤45%. They detected POCD in 37% of patients and cerebral desaturations in 35% of the patients. However, short reductions in rSO2 during surgery is not associated with the incidence of POCD. With regards to the brain injury markers, there were no significant changes in the level of glial fibrillary acidic protein following surgery. This may be explained by a reduced susceptibility of glial cells to ischaemia. On the contrary, there was an elevation in Neuron-Specific enolase in 49% of patients following surgery, but there was no association with POCD. As neuron-specific enolase is also found in erythrocytes and platelets, this elevation may be attributed to haemolysis as a result of blood surface interactions during on-pump cardiac surgery (109, 110). Similarly, in a secondary analysis of 148 patients, Holmgaard et al. (111) did not detect an association between the duration or extent of rSO2 desaturations >10% compared to baseline with the incidence of POCD at discharge, or 3 months post-operatively. The median time of rSO2 > 10% below baseline did not differ between patients with or without POCD at discharge [Difference = 0.0 min, 95% CI: (−3.11, 1.47), *p* = 0.88]. There was no significant association between any neuropsychological test outcomes and the development of POCD. However, a potential confounder may be the more frequent use of noradrenaline in patients with POCD, given noradrenaline is associated with decreased rSO2 (111, 112). Rogers et al. (113) aimed to determine whether a maintaining an absolute rSO2 of >50% or >70% compared to baseline would reduce peri-operative end-organ damage. However, there was no difference in neuropsychological performance in the cognitive domains including attention, verbal memory, motor coordination, psychomotor speed and visuospatial skill. The NIRS group performed better in the verbal fluency test compared to the generic algorithm group (Mean difference 3.73, 95% CI: 1.50–5.96). There was no difference between the groups in regard to the time to intensive care unit discharge, time to hospital discharge, brain injury markers (S100), myocardial injury markers (serum troponin T) or kidney injury markers (113). Furthermore, Semrau et al. (114) demonstrated that pre-operative cognitive impairment is a stronger predictor of POCD compared to intraoperative rSO2 desaturations. They measured neuropsychological function using a standardised assessment known as the Repeatable Battery for Assessment of Neuropsychological Status (RBANS) and the KINARM Standard Tests on the KINARM Endpoint robot. The KINARM robotic test provides quantitative information about cognitive domains, sensory and motor function by asking patients to move the handles and complete visually stimulated tasks. From the RBANS test, there were non-significant impairments in immediate (10 vs. 10%, *p* = 1.00) and delayed memory (12.5 vs. 17.1%, *p* = 0.727), visuospatial (12.5 vs. 9.75%, *p* = 1.00) and language (2.5 vs. 0%, *p* = 1.00) when comparing pre-operative and post-operative results. Similarly, from the KINARM test, there were non-significant impairments on the reverse visually guided reaching task, object hit and avoid tasks, ball on bar tasks and arm positioning match tasks. Notably, pre-operative cognitive performance accounted for 82.2% of variance in post-operative performance (114). Bennett et al. found that their NIRS protocol (commencing intervention when rSO2 dropped below baseline on room air) did not reduce neurocognitive dysfunction post-operatively prior to discharge or at 6 months. However, the length of the ICU stay was significantly shorter in the NIRS group (*p* = 0.026) (101).

#### Benefits of Near-Infrared Spectroscopy

Near-infrared spectroscopy is a cost-effective monitoring modality compared to electroencephalogram and transcranial doppler. These alternatives modalities require additional technical support, specifically for setup and interpretation of the results (49). Secondly, NIRS allows for monitoring of haemodynamic trends, identifying patients at risk of developing POCD, and consequently implementing interventions prior to the occurrence of prolonged or significant cerebral desaturations. This is particularly important in the elderly, as cognitive function naturally declines with age and cardiac surgery may exacerbate this cognitive decline (49, 90, 115). Interventions have included increasing inspired oxygen concentration, altering carbon dioxide levels, increasing mean arterial pressure, and increasing anaesthesia (100). Additionally, the positioning of the NIRS probes over watershed areas such as the anterior and middle cerebral arteries may prevent watershed strokes (116, 117). Furthermore, cerebral desaturations have been associated with greater length of hospital stays and additional financial strains. Fischer et al. demonstrated that a perioperative regional cerebral oxygenation of under 60% for greater than 30 min was associated with an additional 4 days in the hospital, costing an extra $8300 (118).

#### Limitations of Near-Infrared Spectroscopy

Firstly, the cerebral oximetry algorithm assumes that the venous: arterial ratio within the tissue is 70/30. However, this is altered during heart failure and consequently the cerebral oximetry reading may not be accurate. Furthermore, the algorithm also assumes that infrared light travels the same distance in each patient. Hence, in patients with oedema, the infrared light may not reach the vasculature and detect hypoxia in the frontal lobes (49). This is naturally compounded by the inability to detect ischaemia in unmonitored regions such as the posterior circulation. Additionally, false positives have been recorded, with patients being neurologically normal despite periods of cerebral desaturations (119). However, this may reflect optimal intervention shortening the period of intraoperative desaturations. Despite these limitations, to facilitate the integration of NIRS into clinical practice, rSO2 values should be regularly inputted into a patient’s medical records. Furthermore, having a bedside monitor which displays live rSO_2_ values may allow for pre-emptive interventions on the wards to reduce cognitive impairment (90). Lastly, future studies should aim to use standardised intervention parameters when determining when to reverse cerebral desaturations to allow for more robust clinical data.

### Cerebral Microemboli

#### Source of Microemboli

Emboli may cause adverse neurological outcomes through cerebral inflammation, ischaemia, and infarction (120). Microemboli may contribute to POCD by occluding smaller arteries, arterioles and capillaries, whilst macroemboli are thought to contribute to strokes by occluding flow in vessels larger than 200 μm in diameter (121, 122). Emboli may be in the form of thrombi, fat or gas. Lipid emboli are derived from plaque release following aortic manipulation such as cross clamping or canulation/decannulation, or from the cardiotomy suction. Emboli returned through the arterial canula will travel through the aortic arch, reaching sites such as the cerebral vessels (123, 124). In contrast, gaseous emboli may be derived from inadequate deairing of open chamber procedures, introduction by perfusion interventions such as drug delivery or the entrapment of air within the cardiotomy suction (88, 125).

#### Detection of Microemboli

Microemboli are predominantly detected using transcranial doppler ultrasound (TCD) (126). TCD detects high-intensity signals (HITS), a surrogate marker for the number of microemboli (123). HITS are generally detected within the middle cerebral arteries. Currently, TCD cannot determine the composition of the emboli or detect extremely small emboli (<3 mm) (88). Diffuse weighted imaging MRI is the gold standard for detecting ischaemic lesions, but is more costly. However, the use of MRI is limited by several factors. Firstly, MRI cannot provide real-time intraoperative monitoring, whilst a transcranial doppler ultrasound can be used intraoperatively. Moreover, MRI is limited by the logistical and timing requirements, as they cannot be performed perioperatively in contrast to transcranial doppler ultrasounds. Lastly, MRIs are unable to be performed in all patient groups due to contraindications such as having a pacemaker *in situ* (127).

#### Recent Literature

Recent studies have examined the relationship between the embolic load or embolic size and POCD (Table 5). Patel et al. used a novel bubble sizing algorithm to determine the size and volume of bubbles released during cardiac surgery. The number of bubbles and emboli detected were not associated with POCD (*p* = 0.49) (128). The main limitation of this study was the inability to distinguish between the types of emboli. Historically, studies have suggested that reduced embolic load is associated with less POCD (129–131). These conflicting results perhaps suggest that the composition and site of emboli are more important factors in the development of POCD. This supports the notion of “strategic infarcts,” where small lesions or emboli in key brain regions which control cognitive and behaviour may result in more significant cognitive impairment compared to insults in other regions (132). In Wiberg’s study, patients were randomised to low MAP (40–50 mmHg) or high MAP (70–80 mmHg) targets during cardiac surgery. However, there was no relationship between the number of gaseous microemboli and the development of POCD (133). This does not support the washout theory, which states that higher MAP targets during surgery facilitate the clearance of emboli (134).

**TABLE 5:** Studies analysing microemboli and POCD.

Study	Aim	Surgery Type	Study Type	Patient number	Definition of POCD	T1 Cognitive Assessment	T2 Cognitive Assessment	Psychological Assessment Tool	Findings
Halkos et al. (123)	To investigate different operative strategies (aortic clamping) on developing of cerebral emboli as detected with transcranial doppler ultrasound	On-pump or Off-pump CABG	Randomised control trial Off pump group: partial clamp or clampless facilitating device On pump group: single or double clamp	142 Off-pump CABG with partial clamp (*n* = 36) Off-pump CABG with clampless facilitating device (*n* = 36) On-pump CABG with single clamp (*n* = 34) On-pump CABG with double clamp (*n* = 36)	Not specified	Preoperatively	POD30	CNS-VS	No association between cerebral microembolisation and POCD “No difference in cerebral embolic events between on-pump patients undergoing a single clamp-technique and those undergoing a double clamp technique” “More cerebral embolic events occurred in off pump patients when clamp less facilitating device was used compared with partial clamp technique”

Patel et al. (128)	To determine whether high volumes of macrobubbles (>100 uM) entering the brain is associated with an increased risk of cerebral microbleeds or POCD	CABG, valve surgery	Prospective, observational trial	46	Not specified	1–2 weeks before surgery	6–8 weeks postoperatively	TMT A/B GPT WMS WIAS	Total number of emboli (*p* = 0.49) and CPB duration (*p* = 0.99) is not associated with POCD Odds of developing POCD increased by 11% for every year after 64 years of age (*p* = 0.01)

Wiberg et al. (133)	To investigate the association between total number of gaseous micro-emboli in the CPB circuitry and the development of POCD or cerebral infarcts	Cardiac surgery with CPB	Secondary analysis of parallel group randomised trial (PPCI trial) Patients randomised to low MAP (40–50 mmHg) or high MAP target (70–80 mmHg) Number and volume of gaseous micro-emboli passing through inflow CPB canula detected by doppler ultrasound (Bubble Counter Clinical 200) Cerebral infarcts detected by diffusion weighted MRI.	143	Sum of Z score > 1.96, or at least two Z scores for separate tests > 1.96	Day before surgery	Before POD7 or discharge	ISPOCD battery (VLT, CST, SCW, and LDC)	No significant association between number of gaseous microemboli and the development of POCD or cerebral infarctions Significant association between cerebral infarcts and development of POCD 36 (28%) of patients developed POCD and 66 (46%) of patients had cerebral infarctions after 7 days
Bozhinovska et al. (120)	To assess the difference in neurological complications such as POCD following mini-sternotomy vs. mini-thoracotomy using microembolic signals	Aortic valve replacement	Prospective, observational trial Middle cerebral arteries monitored *via* trans-cranial doppler ultrasound	52 Mini-sternotomy (*n* = 25) Mini-thoracotomy (*n* = 27)	Not specified	Day before surgery	Up to 30 days post-operatively	ACE-R	Microembolic signals were not associated with POCD following mini-thoracotomy or mini-sternotomy. Hence, both techniques are comparable. No difference in ACE-R scores between groups (*p* = 0.630).

*ACE-R, addenbrooke’s cognitive examination revised; CABG, coronary artery bypass graft; CNS VS, cns vital signs; CST, concept shifting test; GPT, grooved pegboard test; LDC, letter digit coding; MAP, mean arterial pressure; POCD, postoperative cognitive dysfunction; POD, postoperative day; SCW, stroop colour word interference; T1, pre-operative timepoint for neurocognitive testing; T2, post-operative timepoint for neurocognitive testing; TMT, trail making test; VLT, verbal learning test; WASI, wechsler adult intelligence scale; WMS, wechsler memory test.*

Modification of surgical techniques have been explored to minimise microemboli. Halkos et al. investigated the effect of different aortic clamping strategies on the development of cerebral emboli and POCD. They had four groups: off-pump with partial clamp, off-pump with **a** clampless facilitating device, on-pump with single or double clamp. There was no association between clamping strategies and POCD. They detected more embolic events using TCD in the off-pump group with the clampless facilitating device. There were no differences in embolic events for the on-pump group (123). Bozhinovska et al. (120) explored the difference between mini-sternotomy and mini-thoracotomy for aortic valve replacement on the development of neurological complications. There was no difference in microembolic load between the groups and no association with POCD as detected using TCD. The Addenbrooke’s Cognitive Examination Revised Test score decreased in both groups following surgery (Mini-sternotomy: 85.2 ± 9.6 vs. 82.9 ± 11.4, *p* = 0.012; Mini-thoracotomy: 85.2 ± 9.6 vs. 81.3 ± 8.8, *p* = 0.001). This suggests that the less-invasive mini-thoracotomy approach may be a safe alternative. The main limitation of the mini-thoracotomy approach arises from the possibility of incomplete cardiac deairing at the conclusion of the procedure. The total intraoperative micro-embolic load was associated with the duration of cardiopulmonary bypass.

Issitt et al. demonstrated that a new lipid filtration technique prevents entry of lipid microemboli from the pericardial suction into systemic circulation using a syphon mechanism (*p* < 0.001). This overcomes the current limitations of fat deformability, which allows fats to pass through the CPB filters. The effective filtration of lipid microemboli attenuates the release of neuron-specific enolase, a biomarker for neuronal injury (*p* = 0.013) (135, 136).

### Glycaemic Control

#### Importance of Glycaemic Control During Cardiac Surgery

Optimisation of peri-operative glycaemic control is crucial in cardiac surgery for improving clinical outcomes. Hyperglycaemia is both a cause and consequence of inflammation. Chronic and intraoperative hyperglycaemia is associated with adverse outcomes. Chronic hyperglycaemia increases the risk of developing age related cognitive impairment and dementia (137). This predisposition to neurocognitive impairment may be attributed to a reaction between glycated end-products caused by hyperglycaemia, with the vascular endothelium (138). Conversely, intraoperative hyperglycaemia is associated with arrhythmias, low cardiac output states, postoperative infections, postoperative neurocognitive dysfunctions, and increased mortality in both cardiac surgery patients and critically ill patients in the intensive care unit (139–141). Intraoperative hyperglycaemia may occur due to a stress hyperglycaemic response, which refers to a transient elevation of blood glucose levels in response to illness or injury. Within the cardiac surgery setting, stress hyperglycaemia may occur due to hypothermia, the administration of glucose containing cardioplegia and the administration of heparin (142, 143). Certain physiological processes such as increased substrate availability and renal reabsorption of glucose are enhanced during cardiac surgery, exacerbating the stress hyperglycaemia response (144).

#### Pathophysiological Mechanism

The pathophysiological mechanism linking hyperglycaemia and postoperative cognitive impairment has yet to be fully elucidated. Hyperglycaemia may disrupt the blood brain barrier by damaging pericytes embedded in the basement membrane of blood vessels and increase the susceptibility of these vessels to oxidative stress (145). Disruption of the blood brain barrier facilitates the development of the cerebral oedema, which worsens neurological functioning (146). Additionally, there is an increase in anaerobic glycolysis as a response to activation of the hypothalamic pituitary axis and stress hyperglycaemia during cardiac surgery. This leads to a rise in the concentration of acidic metabolites such as lactate, which contributes to intracellular acidosis. Lactate may cross the disrupted blood brain barrier and contribute to neurocognitive impairment *via* excess increases in intracellular calcium concentration, mitochondrial dysfunction, and protein denaturation (147, 148). Lastly, hyperglycaemia may disrupt the autonomic regulation of cerebral vasculature, delaying neurocognitive recovery following cardiac surgery (148).

#### Association Between Glycaemic Control and Postoperative Cognitive Impairment

Three studies examined the association between glycaemic control and postoperative cognitive impairment following cardiac surgery (Table 6). Kotfis et al. performed a retrospective cohort study to determine whether pre-operative elevation of Haemoglobin A1C (HBA1C) or diabetes was associated with post-operative delirium following cardiac surgery. In an analysis, of 3178 patients, preoperative HBA1C was elevated above 6% in more delirious patients compared to non-delirious patients (44.54 vs. 33.04%; *p* < 0.001). Additionally, patients with delirium were more likely to be diabetic than non-diabetic (42.03 vs. 29.86%; *p* < 0.001) (149). From this, it appears that elevated pre-operative HBA1C and diabetes are risk factors for post-operative delirium following cardiac surgery. However, given the retrospective nature of the study, a causal relationship cannot be established. To overcome this limitation, prospective trials should be performed in the future. Additionally, Zhou et al. conducted an observational trial to examine the effect of impaired fasting glucose on brain injury in patients undergoing on-pump CABG surgery. Patients were followed up based on their pre-operative fasting glucose levels. They found that there were no differences in MMSE scores between the impaired fasting glucose and control groups at 7 days (*p* = 0.704). However, Neuron Specific Enolase (NSE) and S100B concentrations were significantly higher in the impaired fasting glucose group immediately following the termination of CPB, 2 h after the termination of CPB and 24 h postoperatively (148). This suggests that while overt neurocognitive deterioration and postoperative cognitive dysfunction was minimally observed in the impaired fasting glucose group, subclinical manifestations of brain injury may have been present. Scrimgeour et al. (150) conducted a similar observational study, aiming to determine whether perioperative glycaemic control improves neurocognitive decline following cardiac surgery. Patients were followed up based on their pre-operative HBA1C levels (<7 or >7%). Scrimgeour and colleagues found that preoperative elevated HBA1C was not associated with neurocognitive impairment on POD4 (*p* = 0.973). Elevated fasting glucose levels (>126 mg/dL or 7 mmol/l) on the morning of surgery was not associated with neurocognitive impairment on POD4 (*p* = 0.910). This may be potentially explained by a minimal duration of time with metabolic derangements. Scrimgeour et al. also observed that 73% of patients within the trial had a reduction in neurocognitive functioning on POD4 (150). This large incidence of postoperative neurocognitive decline may be attributed to the use of the ‘Repeatable Battery Assessment of Neuropsychological Status,’ which is sensitive in detecting any minor neurocognitive impairment. Scrimgeour and colleagues also compared changes in genetic expression in patients with neurocognitive decline, and without neurocognitive decline following cardiac surgery. They detected an upregulation in anti-inflammatory genes such as Annexin A1 and transforming growth factor B (TGF-B) in patients without neurocognitive dysfunction following surgery. Annexin A1 has anti-inflammatory properties, inhibiting neutrophil migration and inducing the conversion of macrophages into an anti-inflammatory M2 phenotype. Similarly, TGF-B increases the expression of anti-inflammatory cytokine IL-10 and facilitates the conversion of macrophages into an anti-inflammatory M2 phenotype. Future studies should examine whether these gene expression changes are indeed a causative factor in the development of postoperative cognitive dysfunction and whether therapeutic strategies can be developed to target this mechanism.

**TABLE 6:** Studies assessing the effect of glycaemic control on post-operative cognitive impairment following cardiac surgery.

Study	Aim	Surgery Type	Study Type	Patient Number	Definition of POCD	T1 Cognitive Assessment	T2 Cognitive Assessment	Psychological Assessment Tool	Findings
Saager et al. (151)	To determine the effect of tight intraoperative glucose control using hyperinsulinemic-normoglycemic clamp on postoperative delirium following cardiac surgery	Cardiac surgery	Double blind RCT Tight intraoperative glucose control with hyperinsulinemic-normoglycemic clamp (target BSL 80–110 mg/dl or 4.4–6.1 mmol/L). Achieved with constant infusion of insulin and concomitant infusion of 20% dextrose Standard therapy (conventional insulin administration with BSL target <150 mg/dl or 8.3 mmol/L)	198 Hyperinsulinemic clamp (*n* = 93) Standard therapy (*n* = 108)	Not specified	Preoperatively	Not specified	CAM MDAS	Patients in the tight intraoperative glucose control group were 1.89× more likely of developing delirium compared to standard therapy group (RR 1.96; 95% CI: 1.06–3.37; *p* = 0.03) Incidence of delirium was 28% in tight glucose control group and 14% in standard therapy group

Kurnaz et al. (21)	To determine the effect of tightly controlled blood glucose levels during cardiac surgery	On-pump CABG	Double blind RCT Tight glucose control group-maintained blood glucose between 80–120 mg/dl or 4.4–6.7 mmol/L. Liberal control group maintained blood glucose between 80–180 mg/dl or 4.4–10 mmol/L	40 Tight control group (*n* = 20) Liberal control group (*n* = 20)	Drop of one SD from baseline on two or more tests	Day before surgery	1 and 12 week following surgery	Weschler Memory Scale Logical Memory Subtest Clock drawing test Word list generation test Digit span subtest Visuospatial skills test	Tight perioperative glycaemic control following cardiac surgery may prevent persisting cognitive impairment. Incidence of POCD during the first week following surgery was similar between the groups. Persistent POCD was observed in 5 patients (25%) in the tight glucose control group, while no POCD was observed in the liberal control group at 3 months (*p* = 0.047).

Kotfis et al. (149)	To determine whether postoperative delirium following cardiac surgery is associated with diabetes or elevated HBA1C	Major cardiac surgery	Retrospective observational cohort study	3178	Not specified	Not specified	Not specified	CAM	15.8% of patients (502) were diagnosed with postoperative delirium Patients with delirium were more likely to be diabetic (42.03 vs. 29.86%, *p* < 0.001). Preoperative HBA1C was elevated above 6% in more delirious than non-delirious patients (44.54 vs. 33.04%; *p* < 0.001). Risk factors for delirium include older age (>70), ischaemic heart disease, diabetes, arterial hypertension, NYHA3/4, ejection fraction <30% (*p* < 0.01)
Zhou et al. (148)	To determine the effect of impaired fasting glucose on brain injury in patients undergoing cardiac surgery	On-pump CABG	Prospective observational trial Patients divided based on their preoperative fasting glucose levels	50 Impaired fasting glucose (*n* = 25) Control (*n* = 25)	MMSE < 24	Day before surgery	POD7	MMSE	No differences in MMSE scores between the impaired fasting glucose and control groups at 7 days (*p* = 0.704) NSE and S100B concentrations were significantly higher in the impaired fasting glucose group immediately following the termination of CPB, 2 h after the termination of CPB and 24 h postoperatively. Cerebral oxygen extraction ratio and lactic acid expression were significantly higher in the impaired glucose

Scrimgeour et al. (150)	To determine whether perioperative glycaemic control improves neurocognitive decline following cardiac surgery	On-pump CABG or valvular surgery	Prospective observational trial Patients followed up based on their pre-operative HBA1C levels (<7 or >7%)	30	Reduction from baseline	Day before surgery	POD4	RBANS	73% of patients had decrease neurocognitive function at POD4. Preoperative elevated HBA1C was not associated with neurocognitive impairment on POD4 (*p* = 0.973) Elevated fasting glucose levels (>126 mg/dL or 7 mmol/l) on the morning of surgery was not associated with neurocognitive impairment on POD4 (*p* = 0.910) No difference in postoperative neurocognitive function in patients with higher maximum blood glucose levels during CPB (>180 mg/dL or >10 mmol/L; *p* = 0.252) or average glucose levels (>160 mg/dL or >8.9 mmol/l; *p* = 0.639)

*CABG, coronary artery bypass graft; CAM, confusion assessment method; CPB, cardiopulmonary bypass; MDAS, memorial delirium assessment scale; MMSE, mini mental state examination; NSE, neuron-specific enolase; POCD, postoperative cognitive dysfunction; POD, postoperative day; RBANS, repeatable battery assessment of neuropsychological status; RCT, randomised controlled trial; T1, pre-operative timepoint for neurocognitive testing; T2, post-operative timepoint for neurocognitive testing; WMS, wechsler memory test.*

#### Degree of Glycaemic Control and Postoperative Cognitive Impairment

Two studies examined whether tight intra-operative control of blood glucose levels during cardiac surgery may prevent post-operative cognitive impairment (Table 6). Saager et al. (151) compared the effect of tight intra-operative glucose control using a hyperinsulinemic-normoglycemic clamp and standard blood glucose management on postoperative delirium following cardiac surgery. The tightly controlled blood glucose group targetted an intraoperative blood glucose level of 80–110 mg/dL or 4.4–6.1 mmol/L. This was achieved with a constant infusion of insulin and concomitant infusion of 20% dextrose. The standard blood glucose control group targetted an intraoperative blood glucose level less than 150 mg/dL or 8.3 mmol/L, which was achieved *via* conventional insulin administration. Saager et al. observed that patients in the tight intraoperative glucose control were 1.89× more likely of developing delirium compared to the standard therapy group (RR 1.96; 95% CI: 1.06–3.37; *p* = 0.03). The incidence of delirium was also double in the tight glucose control group compared to the standard therapy group (28 vs. 14%) (151). Kurnaz et al. also compared tight and liberal control of intraoperative glucose levels. For the tightly controlled blood glucose group, an insulin infusion was started when blood glucose levels exceeded 120 mg/dL or 6.7 mmol/L. For the liberally controlled blood glucose group, an insulin infusion was started when blood glucose levels exceeded 180 mg/dL or 10 mmol/L. Kurnaz and colleagues observed that the incidence of POCD between the two groups during the first week postoperatively were similar. Additionally, they found that persistent POCD was observed in 5 patients (25%) in the tight glucose control group, while no POCD was observed in the liberal control group at 3 months (*p* = 0.047). This suggests tight intra-operative glycaemic control may prevent persistent cognitive impairment following cardiac surgery (21). However, future studies should aim to use standardised interventions and definitions of tight glycaemic control to establish the most effective method of peri-operative glycaemic control following cardiac surgery.

### Anaesthesia Induced Neurotoxicity

Anaesthesia during cardiac surgery may be induced using intravenous anaesthesia, or as a combination of intravenous and inhalational anaesthesia. Anaesthesia is essential for reducing sensation and inhibiting motor responses during the procedures. This section will discuss the types of anaesthesia agents used in clinical practice, their mechanism of actions, benefits and disadvantages and review the contemporary literature regarding their effect on post-operative cognitive impairment.

#### Volatile Anaesthetic Agents

Volatile anaesthetics exist in the liquid state at room temperature, before being vaporised into gas for absorption and distribution through the alveoli and pulmonary circulation. There are three main groups of volatile anaesthetics including halogenic ethers, alkanes, and gaseous agents. The halogenic ethers include isoflurane, sevoflurane, enflurane and desflurane. This group is the most common used in cardiac surgery. Secondly, the main alkane volatile anaesthesia agent is halothane. Halothane is the main non-ether anaesthetic in use today in modern clinical practice. Lastly, the gaseous volatile anaesthetic agents include nitrous oxide and xenon (152, 153). The specific mechanism of action of inhaled anaesthetic agents is unknown. However, it is known that they broadly potentiate the inhibitory, type A Gamma-aminobutyric acid (GABA_*A*_) receptors within the central nervous system and two pore domain K^+^ channels to modulate neurotransmission. Additionally, the gaseous inhaled agents function by inhibiting the N-methyl-D-aspartate (NMDA) receptors (152, 154).

The use of inhaled halogenic ether anaesthetic agents such as isoflurane and sevoflurane have been demonstrated to be cardioprotective during cardiac surgery. Sevoflurane enhances the post-operative recovery of the stunned myocardial contractility, reduces myocardial ischaemia and the extent of reperfusion injury by scavenging oxygen free radicals (155, 156). These beneficial effects are a result of alterations in intra-cellular signalling pathways such as *via* the G-protein coupled-receptors, changes in mitochondrial function as well as potassium channel functioning (157). Recent studies and meta-analyses have reported conflicting evidence on the renal and hepato-protective properties of volatile anaesthetics (158–160). Further human studies are required to elucidate the potential beneficial effects of volatile anaesthetics on other organ systems.

Whilst inhaled anaesthetic agents are essential for cardiac surgery, their use have been implicated the development in post-operative complications such as POCD and the development of Alzheimer’s Dementia. There are numerous speculated molecular mechanisms regarding their potential neurotoxic nature. Pre-clinical studies from cell cultures and animal models have demonstrated that volatile anaesthesia may be cytotoxic, alter neuronal morphology, and increase caspase-3 expression, culminating in neuronal apoptosis. Additionally, there may be formation, oligomerisation and deposition of amyloid in response to anaesthesia (161). However, the literature has not shown a clear association between the exposure to general anaesthesia and the development of Alzheimer’s disease (162).

#### Intravenous Anaesthesia

Intravenous anaesthesia can be used for both the induction and maintenance of general anaesthesia. Examples of intravenous anaesthesia includes propofol, dexmedetomidine, ketamine, barbiturates, and benzodiazepine (163). There has been significant research interest in the use of propofol and dexmedetomidine during cardiac surgery within the last few years. Propofol is a phenol compound with a rapid onset and short duration of action. Mechanistically, it primarily functions by potentiating the inhibitory GABA_*A*_ neurotransmitter response. Propofol also inhibits NMDA receptors, which prevents calcium ion influx and consequently reduces the likelihood of cytotoxic intracellular calcium overload. Furthermore, propofol may inhibit neuronal apoptosis through the modulation of cell survival and apoptotic proteins such as Bcl-2 and inhibits caspase-3. The use of propofol is associated with benefits such as ease of patient waking with minimal secretions, and anti-emetic properties. However, care must be taken when using propofol to prevent dose dependent hypotension and respiratory depression (164, 165). Dexmedetomidine is a selective α-2 adrenergic receptor agonist, which has been increasingly used. Section 3.6 provides a detailed discussion on the use of dexmedetomidine to reduce postoperative cognitive impairment.

#### Volatile Anaesthesia vs. Intravenous Anaesthesia

The European Association for Cardio-Thoracic Surgery as well as the American College of Cardiology and American Heart Association have recently released their guidelines stating a preference for using volatile anaesthesia during coronary artery bypass graft surgery (166, 167). However, the superiority of volatile anaesthesia over total intravenous anaesthesia for reducing poor outcomes is unclear. Four trials and two systematic reviews were identified comparing volatile and intravenous anaesthesia (Table 7).

**TABLE 7:** Studies assessing the effect of anaesthesia on cognitive impairment following cardiac surgery.

Study	Aim	Surgery Type	Study Type	Patient Number	Definition of POCD	T1 Cognitive Assessment	T2 Cognitive Assessment	Psychological Assessment Tool	Findings
Tang et al. (171)	To compare the effect of propofol vs. sevoflurane on cognitive impairment following on-pump cardiac surgery	On-pump cardiac surgery	RCT Sevoflurane administered to maintain end expiratory and end-effluent concentrations of 1–3% Propofol was infused to reach a serum concentration of 0.5–2.0 ug/kg/min with a BIS index of 40–55 intraoperatively	110 Propofol (*n* = 55) Sevoflurane (*n* = 55)	Not specified in study	Day before surgery	12 and 24 h postoperatively	MMSE	MMSE scores (Mean ± SD) were higher in the propofol group compared to the sevoflurane group when measured 24 h post-operatively (28.74 ± 4.53 vs. 24.30 ± 3.77; *p* < 0.05) Serum levels of NSE, S100B and MMP-9 were lower in the propofol group compared to the sevoflurane group at 6, 12, and 24 h post-operatively (*p* < 0.05) Incidence of POCD was significantly lower at 12 and 24 h postoperatively in the propofol group compared to the sevoflurane group (*p* < 0.05). POCD incidence in Sevoflurane group at 12 h: 12 patients (21.82%) POCD incidence in propofol group at 12 h: 5 patients (9.09%) POCD incidence in sevoflurane group at 24 h: 14 patients (25.45%) POCD incidence in propofol group at 24 h: 6 patients (10.91%)

Shi et al. (169)	To compare the effects of different maintenance methods on postoperative outcomes, inflammation, and haemodynamic stability	Off-pump CABG	RCT Control group (propofol 3 mg/kg/hr and fentanyl 5 ug/kg/hr for maintenance of anaesthesia) Observation group (Sevoflurane 3–4% concentration titrated to a BIS value for maintenance of anaesthesia)	94 Control group (*n* = 47) Observation group (*n* = 47)	Reduce in score compared to baseline	Day before surgery	Day after surgery	MMSE MoCA	A reduction in MMSE and MoCA scores postoperatively was lower in the observation group (sevoflurane) compared to the control group (*p* < 0.005). Larger increase in inflammatory markers (CRP, TNF-a, IL-6) in the control group compared to the observational group following surgery (*p* < 0.05) No significant difference in haemodynamic parameters including heart rate, mean arterial pressure, systemic vascular resistance index and cardiac index between the groups (*p* > 0.05) Intraoperative serum levels (Mean ± SD) of Malondialdehyde were lower in the observational group compared to the control group (3.48 ± 1.02 vs. 6.24 ± 1.07; *p* < 0.001) No difference in the incidence of adverse drug reactions between the groups including nausea/vomiting, hypoxaemia, postoperative agitation or delay in waking (*p* > 0.05)
Landoni et al. (172)	To determine whether volatile anaesthesia vs. total intravenous anaesthesia for cardiac surgery would improve clinical outcomes	Both on-pump and off-pump CABG	RCT Volatile vs. total intravenous anaesthesia	5400 Volatile anaesthesia (*n* = 2709) Total intravenous anaesthesia (*n* = 2691)	“Cognitive deterioration that exceeds a ‘normal’ age- and time-attributed cognitive decline and which is caused by negative consequences of peri-operative condition” Statistical definition not specified	Not specified	Not specified	Not specified	No significant difference between the volatile and total intravenous anaesthesia groups with respect to all-cause mortality at 1 year (RR = 0.94, 95% CI: [0.69–1.29], *p* = 0.71) No difference in the incidence of post-operative cognitive impairment between the groups (Volatile: 1.1%, total intravenous: 1.2, 95% CI: 0.56–1.48)

Wang et al. (174)	To compare the effect of propofol vs. sevoflurane on chronic post-surgical pain and cognitive function after cardiac surgery in Chinese patients >65 years old	On-pump CABG	RCT Propofol infused at 3–8 mg/kg/h Sevoflurane administered to achieve end tidal concentration of 0.5–2	220 Sevoflurane (*n* = 110) Propofol (*n* = 110)	Not specified in study	Day before surgery	Not specified in study	MMSE	Patients who received propofol had a smaller reduction in MMSE scores (Mean score) following cardiac surgery compared to patients who received sevoflurane (64 vs. 59, respectively). However, significance was not achieved

*BIS, bispectral index; CABG, coronary artery bypass graft; IL-6, Interleukin-6; MMSE, mini mental state examination; MOCA, montreal cognitive assessment; MMP-9, matrix metalloproteinase 9; NSE, neuron specific enolase; POCD, postoperative cognitive dysfunction; POD, postoperative day; RCT, randomised controlled trial; T1, pre-operative timepoint for neurocognitive testing; T2, post-operative timepoint for neurocognitive testing; tMT, trail making test; TNF-a, tumour necrosis factor-alpha; WAIS, wechsler adult intelligence scale; 6-CIT, six-item cognitive impairment test.*

Chen et al. conducted the first systematic review and meta-analysis to compare the neuroprotective effects of inhalational anaesthesia compared to total intravenous anaesthesia (such as propofol and sodium thiopental) in cardiac surgery with cardiopulmonary bypass (168). The inhalational anaesthesia included within the systematic review consisted of isoflurane, sevoflurane and desflurane. Conversely, the intravenous anaesthesia included within the study consisted of propofol, thiopental, midazolam, and ketamine. From six studies involving 230 patients, the serum level of S100B (Weighted Mean Difference; 95% CI) was significantly lower in the inhalational anaesthesia group compared to the total intravenous anaesthesia following cardiopulmonary bypass (−0.41, 95% CI [−0.81, −0.01], *p* = 0.05) and 24 h post-operatively (−0.32, 95% CI [−0.59, −0.05], *p* = 0.02). Additionally, from three studies involving 110 patients, the MMSE score (Weighted Mean Difference; 95% CI) was significantly higher in the inhalational anaesthesia group compared to the total intravenous anaesthesia group at 24 h post-operatively (1.87, 95% CI [0.82, 2.92], *p* = 0.002). These results highlight the potential superiority of inhalational anaesthesia compared to total intravenous anaesthesia for neuroprotection in patients undergoing cardiac surgery. Shi et al. (169) compared the combination of propofol and fentanyl to sevoflurane for maintenance of anaesthesia in off-pump CABG. The reduction in MMSE and MoCA scores postoperatively was lower in the sevoflurane group compared to the propofol/fentanyl group (*p* < 0.005). Additionally, there was a larger increase in inflammatory markers (CRP, TNF-a, IL-6) in the propofol/fentanyl group compared to the sevoflurane group following surgery (*p* < 0.05). These results indicate that sevoflurane, compared to propofol/fentanyl attenuates the inflammatory response during cardiac surgery. This may have contributed to a lower reduction in MMSE and MoCA scores post-operatively in the sevoflurane group. Furthermore, the intraoperative serum levels (Mean ± SD) of Malondialdehyde were lower in the sevoflurane group compared to the propofol/fentanyl group (3.48 ± 1.02 vs. 6.24 ± 1.07; *p* < 0.001) (169). Malondialdehyde is a metabolite within the oxidative stress pathway and reflects the level of oxidative stress (170). The lower levels of intraoperative malondialdehyde within the sevoflurane group reaffirms the current mechanistic understanding that volatile anaesthetics can scavenge free oxygen radical and attenuate oxidative stress.

Tang et al. compared the effect of sevoflurane vs. propofol on cognitive impairment following on-pump cardiac surgery (171). Sevoflurane was administered to maintain end expiratory and end-effluent concentrations of 1–3%, whilst propofol was infused to reach a serum concentration of 0.5–2.0 ug/kg/min with a BIS index of 40–55 intraoperatively. They observed a lower incidence of POCD at 12 and 24 h post-operatively in the propofol group compared to the sevoflurane group. The incidence of POCD was significantly lower at 12 and 24 h postoperatively in the propofol group compared to the sevoflurane group (*p* < 0.05). At 12 h, the incidence of POCD in the sevoflurane group was 21.82%, whilst it was 9.09% in the propofol group. At 24 h, the incidence of POCD in the sevoflurane group was 25.45%, whilst it was 10.91% in the propofol group. Additionally, the MMSE scores (Mean ± SD) was higher in the propofol group compared to the sevoflurane group when measured 24 h post-operatively (28.74 ± 4.53 vs. 24.30 ± 3.77; *p* < 0.05). Furthermore, serum levels of NSE, S100B and MMP-9 were lower in the propofol group compared to the sevoflurane group at 6, 12, and 24 h post-operatively (*p* < 0.05). Taken together, this study depicts a strong case for the use of propofol over volatile anaesthesia to reduce the incidence of POCD following during cardiac surgery.

Landoni et al. (172) conducted the MYRIAD trial, a multi-centred RCT spanning 36 centres in 13 countries to compare the clinical outcomes in patients receiving volatile anaesthesia or total intravenous anaesthesia undergoing CABG surgery. Whilst there was no strict inhalational anaesthesia protocol, the authors had recommended the use of cardioprotective strategies for patients receiving volatile anaesthesia, including achieving a minimum alveolar concentration of one for at least 30 min, the wash out of volatile anaesthesia prior to initiating CPB and performing at least three wash in/wash out periods. At least one of these cardioprotective strategy was applied in 97.4% of patients receiving volatile anaesthesia, whilst all three strategies were applied in 9.9% of patients receiving volatile anaesthesia. With a sample size of 5400 patients, there was no significant difference in all-cause mortality at 1 year postoperatively between the volatile and total intravenous anaesthesia groups (RR = 0.94, 95% CI: [0.69–1.29], *p* = 0.71). Whilst Landoni and colleagues did not specifically provide a statistical definition for POCD or state how they measured POCD, they did not find a difference in the incidence of POCD between the groups (Volatile Anaesthesia: 1.1% incidence, Total Intravenous Anaesthesia: 1.2% Incidence, 95% CI [0.56–1.48]) (172). It should be noted that some investigators decided to co-administer propofol during the induction of anaesthesia. Studies have shown that the co-administration of propofol may reduce the beneficial effect of volatile anaesthesia, which may explain the observed non-significant result (173). Future studies should aim to use a standardised anaesthesia protocol which reflect clinical practices. Wang et al. (174) compared the effect of sevoflurane and propofol on chronic post-surgical pain following cardiac surgery in Chinese patients aged >65 years. Propofol was infused at 3–8 mg/kg/hr, whilst sevoflurane was administered to achieve an end tidal concentration of 0.5–2. They found that patients who received propofol had a smaller reduction in MMSE scores (Mean score) following cardiac surgery compared to patients who received sevoflurane (64 vs. 59, respectively). However, significance was not achieved (174). Jiao et al. (175) conducted a systematic review and meta-analysis to compare the effect of volatile anaesthetics and total intravenous anaesthesia in patients undergoing coronary artery bypass grafting. A total of 89 studies comprising of 14,387 patients were included in the meta-analysis. From the eight studies within the meta-analysis reporting on post-operative cognitive impairment, there was no significant difference between volatile anaesthesia and total intravenous anaesthesia (RR = 1.20, 95% CI: 0.74–1.94, *p* = 0.46). It should be noted that quality of the studies included for this outcome were rated as low by the authors. From the forty-four studies which examined operative mortality, there was no significant difference between volatile anaesthesia and total intravenous anaesthesia (RR = 0.92, 95% CI: 0.68–1.24, *p* = 0.59). Five studies did not find a significant difference in mortality at 1 year when comparing volatile and total intravenous anaesthesia (RR = 0.64, 95% CI: 0.32–1.26, *p* = 0.19). Forty-three studies demonstrated that the length of stay in the intensive care unit was significantly shorter in the volatile anaesthesia group compared to the total intravenous anaesthesia group (Mean Difference = −4.14 h, 95% CI: −5.63–−2.66, *p* < 0.00001). Similarly, thirty-four studies revealed that the length of stay in hospital was also shorter in the volatile anaesthesia group compared to the total intravenous anaesthesia group (Mean Difference = 01.22 days, 95% CI: −1.81–−0.62 days, *p* < 0.0001) (175).

### Dexmedetomidine

Dexmedetomidine is frequently used for anaesthesia and within the intensive care setting, acting as a selective α-2 adrenergic receptor agonist. There has been significant interest in dexmedetomidine as it may reduce POCD following cardiac surgery. Dexmedetomidine has numerous therapeutic uses, acting as a sedative, anxiolytic, anti-inflammatory, and analgesic. It has also been shown to inhibit sympathetic outflow and provide neuroprotection (176). Firstly, the anti-inflammatory properties of dexmedetomidine are reflected by a significant decrease of serum cytokines IL-6, IL-8, and TNF-a following major surgery in humans (177, 178). This may be attributed to a downregulation of TLR receptors, as observed in an aged mice model (179). Dexmedetomidine also increases the transcription of brain derived neurotrophic factors, anti-apoptotic proteins and decrease nerve sensitivity to glutamate. These gene expression changes, in combination with the previously mentioned anti-inflammatory effects, produce a neuroprotective effect (176).

The analgesic properties arise from the inhibition of substance p and activation of α-2B adrenoceptors within the dorsal horn. These analgesic effects are achieved with minimal concerns of respiratory depression, unlike traditional opioids (180). On the contrary, the activation of α-2A adrenoceptors inhibits central sympathetic outflow from the locus coeruleus, culminating in a reduction of catecholamine release (181). This in turn reduces the heart rate and systemic vascular resistance. Additionally, the sedative and anxiolytic effects also arise from interactions within the locus coeruleus. These therapeutic effects have stirred significant interest into the potential use of dexmedetomidine for reducing postoperative cognitive impairment following cardiac surgery.

Six recent studies analysed the effect of dexmedetomidine on postoperative cognitive functioning following cardiac surgery (Table 8). Three studies supported the use of dexmedetomidine. Gong et al. demonstrated that MMSE and MoCA scores were significantly higher in the dexmedetomidine group compared to the control group (*p* < 0.05 and *p* < 0.01, respectively) at 1 day postoperatively following on-pump CABG. They also observed that the use of dexmedetomidine is associated with a quicker anaesthesia recovery time, and a reduced duration of mechanical ventilation compared to the control group (*p* < 0.001) (178). Gao et al. examined the effect of dexmedetomidine vs. control on postoperative cognitive function and neuroglobin expression following minimally invasive off-pump CABG. It should be noted that off pump CABG is prone to intra-operative oxygen desaturation and associated with POCD, given the requirement for single lung ventilation (182, 183). The use of dexmedetomidine was associated with a lower incidence of POCD at POD7 and POD30 (*p* < 0.05) and higher MMSE scores (Mean ± SD) at POD7 (24.8 ± 1.3 vs. 20.5 ± 1.4; *p* < 0.001) and POD30 (28.3 ± 1.5 vs. 24.2 ± 1.2; *p* < 0.001). They also detected an association between dexmedetomidine and increased expression of neuroglobin at 6 h post single lung ventilation and POD1 (*p* < 0.01). Neuroglobin is a hypoxia inducible protein which is highly expressed in the frontal lobes and hypothalamus. It has a high affinity for oxygen, increasing oxygen delivery to the brain (183, 184). These results suggest that neuroglobin may be neuroprotective for postoperative cognitive impairment. While the current literature surrounding the role of neuroglobin is conflicting, these promising results suggest further study into dexmedetomidine and neuroglobin is warranted (184). Rajaei et al. compared the effects of dexmedetomidine and midazolam on postoperative cognitive functioning following on-pump CABG. The use of midazolam was associated with significantly lower WMS scores (Mean ± SD) compared to dexmedetomidine at POD30 (87.6 ± 14.30 vs. 103.53 ± 19.93; *p* < 0.05). However, there was no difference in MMSE scores (*p* = 0.394) (185).

**TABLE 8:** Studies analysing the effect of dexmedetomidine on postoperative cognitive functioning following cardiac surgery.

Study	Aim	Surgery Type	Study Type	Patient Number	Definition of POCD	T1 Cognitive Assessment	T2 Cognitive Assessment	Psychological Assessment Tool	Findings
Gong et al. (178)	To determine the effect of dexmedetomidine on postoperative cognitive function in patients undergoing CABG	On-pump CABG	RCT Dexmedetomidine vs. placebo (saline) Dexmedetomidine 1 ug/kg administered during first 10 min, followed by 0.2 ug/kg infusion until the end of the procedure	80	MMSE < 27 MoCA < 26	Preoperatively	POD 1, 3, 7	MMSE MoCA	MMSE and MOCA scores in the dexmedetomidine group were significantly higher than the scores in the control group (*p* < 0.05 and *p* < 0.01 respectively) Quicker anaesthesia recovery time and lower duration of mechanical ventilation time in the dexmedetomidine group compared to the control group (*p* < 0.001) Lower levels of cortisol, adrenaline and noradrenaline in the dexmedetomidine group compared to the control group at the end of surgery (*p* < 0.001)

Rajaei et al. (185)	To compare the effects of dexmedetomidine and midazolam on postoperative cognitive impairment following CABG	On-pump CABG	Double Blind RCT Midazolam (0.05–0.1 mg/kg) vs. Dexmedetomidine (1 ug/kg) administered as induction of anaesthesia	42	Not specified	Day before surgery	POD 5 and POD30	MMSE WMS	Midazolam group had lower WMS scores (Mean ± SD; 87.6 ± 14.30) and increased cognitive impairment compared to dexmedetomidine group (103.53 ± 19.93; *p* = 0.05) 30 days following surgery. No difference in MMSE scores between the groups postoperatively (*p* = 0.394).

Metry et al. (189)	To compare the effect of dexmedetomidine vs. propofol on postoperative cognitive impairment and rSO2 following cardiac surgery	On-pump CABG	Double blind RCT Propofol (0.3–4 mg/kg/hr) vs. dexmedetomidine (0.2–0.7 mcg/kg/h) infusion following cannulation to the CPB. Drugs ceased when off pump.	50	Decrease in MMSE by 2 or more points compared to pre-operative scores	Day before surgery	1 h following extubation and POD7	MMSE	Pre-operative mean MMSE in both groups: 30 Non-significant decrease in MMSE scores in both groups 1 h post extubation (Mean ± SD; Propofol 23.3 ± 0.408; Dexmedetomidine 23.2 ± 0.41). MMSE performance normalised by POD7 (Propofol 30; Dexmedetomidine 30) Comparison between the groups was not conducted.
Kang et al. (186)	To compare the effect of dexmedetomidine + isoflurane vs. isoflurane anaesthesia on brain injury	On-pump valve replacement surgery	Single blind RCT Dexmedetomidine given as bolus infusion at 0.6 ug/kg, then as a continuous 0.2 ug/kg/h following intubation. Isoflurane given through oxygenator of CPB, targetting BIS of 40–50	97 Dexmedetomidine + Isoflurane (*n* = 50) Isoflurane (*n* = 47)	Decrease of scores more than 30% compared to pre-operative scores	Day before surgery	POD7	ASEM	No significant difference in ASEM score (Mean ± SD) between intervention and control group following surgery (13.69 ± 6.8 vs. 14.0 ± 6.5; *p* = 0.714). Dexmedetomidine group had significantly lower levels of GFAP than isoflurane alone group following CPB off (0.70 ± 0.05 vs. 0.81 ± 0.04; *p* = 0.033). Dexmedetomidine group had significantly lower levels of MMP-9 than isoflurane alone group following CPB off (90.5 ± 15.3 vs. 118.2 ± 17.1; *p* = 0.007) and on POD1 (87.2 ± 12.2 vs. 102.9 ± 11.5; *p* = 0.036)

Gao et al. (183)	To determine the effect of dexmedetomidine on postoperative cognitive function and neuroglobin expression following minimally invasive CABG	Minimally invasive off-pump CABG	RCT Dexmedetomidine vs. control (normal saline) Dexmedetomidine given as bolus infusion at 0.6 ug/kg 15 min prior to induction of anaesthesia, then as a continuous 0.2 ug/kg/h until the end of intubation	40 Dexmedetomidine (*n* = 20) Control (*n* = 20)	MMSE < 27	Day before surgery	POD7, POD30, POD90	MMSE	POCD incidence significantly lower in the dexmedetomidine group at POD7 and POD 30 (*p* < 0.05) MMSE scores (Mean ± SD) of dexmedetomidine group significantly higher compared to control group at POD7 (24.8 ± 1.3 vs. 20.5 ± 1.4; *p* < 0.001) and POD30 (28.3 ± 1.5 vs. 24.2 ± 1.2; *p* < 0.001) NgB expression significantly higher in the dexmedetomidine group compared to the control group at 6 h post single lung ventilation and at POD1 (*p* < 0.01).

*ASEM, anti-saccadic eye movement test; BIS, bispectral index; CABG, coronary artery bypass graft; CAM, confusion assessment method; CRT, choice reaction test; GFAP; glial fibrillary acidic protein; MMSE, mini mental state examination; MOCA, montreal cognitive assessment; MMP-9, matrix metalloproteinase 9; NgB, Neuroglobin B; NSE, neuron specific enolase; POCD, Postoperative cognitive dysfunction; POD, postoperative day; PRM, pattern recognition memory test; rSO2, regional cerebral oxygen saturation; RCT, randomised controlled trial; SCWI, stroop colour word interference; SSP, spatial span test; SRM, spatial recognition memory test; T1, pre-operative timepoint for neurocognitive testing; T2, post-operative timepoint for neurocognitive testing; VLT, verbal learning and retention test; WMS, wechsler memory scale.*

Two studies had conflicting results regarding the use of dexmedetomidine for postoperative cognitive impairment. Kang et al. examined whether the effect of dexmedetomidine and isoflurane compared to isoflurane alone on brain functioning in patients undergoing on-pump valve replacement surgery. There was no difference in ASEM score (Mean ± SD) between intervention and control group following surgery (13.69 ± 6.8 vs. 14.0 ± 6.5; *p* = 0.714). However, the group with dexmedetomidine had significantly lower levels of glial fibrillary acidic protein (GFAP) and MMP-9 following surgery compared to the control group (*p* = 0.033 and *p* = 0.036, respectively) (186). GFAP is a brain specific intermediate protein. Serum levels of GFAP > 0.49 ng/mL are considered pathological and may be indicative of ischaemic or haemorrhagic brain injury. Conversely, MMP-9 expression is increased following brain injury and ischaemia (187). Therefore, these results suggest that dexmedetomidine may reduce the extent of brain insult following cardiac surgery. Xiong et al. (188) conducted a systematic review on the effects of dexmedetomidine on perioperative neurocognitive disorders. With data from 3610 patients collated from 24 studies, they detected no significant difference in the incidence of POCD between dexmedetomidine and the control group (OR: 0.47, 95% CI: 0.22–1.03, *p* = 0.060). It should be noted that numerous studies may have used an insufficient maintenance dose of dexmedetomidine, ranging between 0.2–0.5 ug/kg/hr of dexmedetomidine. In contrast, there was a significant reduction in the incidence of postoperative delirium in the dexmedetomidine group compared to the control group (OR: 0.59, 95% CI: 0.43–0.82, *p* = 0.001) (188).

One study did not support the use of dexmedetomidine for postoperative cognitive dysfunction. Metry et al. examined the effect of dexmedetomidine compared to propofol on postoperative cognitive impairment following cardiac surgery (189). They reported a non-significant reduction in MMSE scores in both groups 1 h following extubation (Mean ± SD; Propofol 23.3 ± 0.408; Dexmedetomidine 23.2 ± 0.41). A direct comparison between the groups was not performed.

## Future Directions

Large, multi-centred, double blinded randomised controlled trials are required to produce higher level evidence in order to evaluate the effectiveness of interventions in reducing POCD. Many trials within the literature have shown benefits for interventions, but the sample sizes were extremely small and within a specific population. This has reduced external validity. Currently, investigators are assessing POCD with a battery of neuropsychological tests which they believe most effectively analyse the cognitive domain of interest. However, this may lead to extensive testing and a drop in concentration or dis-engagement. Thus, the development of a validated neuropsychological battery specifically assessing for POCD following cardiac surgery is necessary. To reduce the learning effect from multiple follow ups, alternative forms of the neuropsychological tests should be used at each encounter. Additionally, there is little consensus on the statistical assessment of POCD. This must be addressed to reduce the heterogeneity in the detection of POCD. Given the recent nomenclature recommendations by Evered et al. (5), future studies should adopt the terms delayed neurocognitive recovery (for cognitive impairment up to 30 days post-operatively) and post-operative mild/major neurocognitive disorders (for cognitive impairment up to 1 year post-operatively). Studies should also adopt follow up times which reflect the duration of these cognitive impairment episodes (i.e., 30 days and 1 year). The combination of a standardised neuropsychological battery for cardiac surgery, a consistent statistical definition for POCD and similar follow-up times between studies will hopefully lead to a more accurate rate of detecting POCD.

Patient risk factors for POCD should be recorded prior to surgery. The strongest risk factors have been shown to be advanced age and lower education attainment. This would allow clinicians to identify patients most at risk of POCD and monitor them closely. The PPCI trial by Vedel et al. (93) has suggested that patients may potentially receive diffusion weighted MRI scans prior to surgery and receive scans post-operatively to identify the development of new cerebral lesions. Given this is a relatively novel technique, more data is required before it can be introduced into clinical practice. Transcranial doppler may also be used for intra-operative monitoring of cerebral blood flow and autoregulation. This has been introduced in several centres already.

There is significant interest in identifying effective therapeutic strategies to attenuate the pathophysiological mechanisms leading to POCD following cardiac surgery. Given the use of low dose dexamethasone (0.1 mg/kg) may be effective in reducing POCD in the long term, future studies assessing the efficacy of dexamethasone should use this dose as their intervention to confirm or refute the finding. Studies on intra-operative blood pressure management appear to be inconclusive. The use of NIRS to detect cerebral desaturations and enable quick intervention is promising. Whilst the evidence is conflicting, the use of NIRS is cost-effective and can be feasibly integrated into clinical practice if further positive data emerges. Additionally, there is limited data on intra-operative glycaemic control during cardiac surgery and suggest there should be some focus on this area. Furthermore, there are limited human studies assessing the mechanism of anaesthesia induced neurotoxicity. Of particular interest is understanding whether anaesthesia truly accelerates the Alzheimer’s pathophysiological process and how anaesthesia ties into POCD. This may potentially alter anaesthetic practice if agents are identified which strongly contribute to POCD.

## Conclusion

This review provides a contemporary overview on postoperative cognitive impairment following cardiac surgery and examines the main pathophysiology mechanisms contributing to such including inflammation, cerebral hypoperfusion, cerebral microemboli, glycaemic control and anaesthesia induced neurotoxicity. Therapeutic areas of interest targetting these mechanisms were examined. The efficacy of dexamethasone in attenuating the inflammatory response is currently questionable. The use of NIRS to monitor for cerebral desaturations and cerebral perfusion appears to be beneficial by allowing clinicians to correct desaturations. However, further studies using similar thresholds for initiating interventions are required. Recent studies demonstrated that emboli size and a higher target MAP during cardiac surgery were not associated with cognitive impairment. Nevertheless, the modification of surgical techniques to reduce the occurrence of emboli and POCD have been promising.

## Author Contributions

TV and JS conceptualised and designed the study. TV collected and analysed the data and produced the first draft of the manuscript. Both authors contributed to subsequent drafts of the manuscript, including editing, and refining of the final manuscript and approved the final version of the manuscript for submission.

## Conflict of Interest

The authors declare that the research was conducted in the absence of any commercial or financial relationships that could be construed as a potential conflict of interest. The handling editor declared a shared affiliation with the authors, JS at the time of review.

## Publisher’s Note

All claims expressed in this article are solely those of the authors and do not necessarily represent those of their affiliated organizations, or those of the publisher, the editors and the reviewers. Any product that may be evaluated in this article, or claim that may be made by its manufacturer, is not guaranteed or endorsed by the publisher.

## Abbreviations

CABG: coronary artery bypass graft
BBB: blood brain barrier
CPB: cardiopulmonary bypass
CAM: confusion assessment method
COX: cyclooxygenase
CST: Concept Shift Test
DAMP: damage associated molecular patterns
DSM-V: Diagnostic and Statistical Manual of Mental Disorders 5th edition
DWI: diffusion weighted imaging
GABA: γ -aminobutyric acid
GPT: Grooved Pegboard Test
HBA1C: Haemoglobin A1C
HITS: high intensity signals
HMGB1: high-mobility group 1
HVLT: Hopkins Verbal Learning Test
IL: interleukin
MAP: mean arterial pressure
MMSE: mini-mental state examination
MMP: matrix metalloproteinases
MoCA: Montreal Cognitive Assessment Scale
NF-KB: nuclear factor kappa beta
NIRS: near-infrared spectroscopy
NMDA: N-methyl -D-aspartate
NCD: neurocognitive disorder
NSE: neuron specific enolase
POCD: post-operative cognitive dysfunction
POD: postoperative day
RAVLT: Rey Auditory Verbal Learning Test
RBANS: Repeatable Battery for the Assessment of Neuropsychological Status
rSO_2_: regional cerebral oxygen saturation
TCD: transcranial doppler ultrasound
TNF-a: tumour necrosis factor a
TGF-B: transforming growth factor B
TMT: Trail Making Test
WMS: Wechsler Memory Scale.
